# Proteomic analysis of plasma and extracellular vesicles from subjects with impaired vascular health

**DOI:** 10.1042/BSR20260067

**Published:** 2026-08-25

**Authors:** Yibo Gao, Lasse G. Lorentzen, Emily M. Martin, Federica Genovese, Gernot Faustmann, Johanna Grabher-Moser, Beate Tiran, Brigitte M. Winklhofer-Roob, Michael J. Davies

**Affiliations:** 1 Department of Biomedical Science, University of Copenhagen, Copenhagen, Denmark; 2 Department of Vascular Surgery, Heart Center, University Hospital Copenhagen - Rigshospitalet, Copenhagen, Denmark; 3 Nordic Bioscience A/S, Herlev, Denmark; 4 Institute of Molecular Biosciences, University of Graz, Humboldtstrasse 50, 8010 Graz, Austria; 5 Clinical Division of Nephrology, Department of Internal Medicine, Medical University of Graz, Austria; 6 Clinical Institute of Medical and Chemical Laboratory Diagnostics, Medical University of Graz, Austria

**Keywords:** biomarkers, cardiovascular disease, extracellular matrix, Extracellular vesicles, protein degradation, proteomics

## Abstract

Cardiovascular diseases are the leading cause of mortality worldwide, with atherosclerosis and formation of arterial plaques being a major underlying cause. Rupture or erosion of the plaque fibrous cap can result in thrombus formation, arterial occlusion, and a stroke or myocardial infarction. Plaque changes, and endothelial cell barrier leakiness, may result in material leakage, including proteins and fragments into plasma either directly or in extracellular vesicles (EVs). Here, we report comparative LC-MS/MS analyses of plasma-derived EVs and plasma from subjects with impaired vascular status and healthy controls. Analysis of plasma-derived EVs detected 7228 peptides and 763 proteins, with 87 proteins being differentially abundant with these including arterial-cell species. Sub-group analysis based on biological sex showed no statistically significant differences for males, whereas females exhibited eight differentially expressed proteins. Subject age effects were minimal. Plasma analysis detected 4366 peptides and 497 proteins, with 188 proteins being significantly altered in abundance between the groups. Subgroup analysis by biological sex revealed 103 differentially expressed proteins in females and 84 in males. No differences were detected in specific collagen fragments. Gene Set Enrichment Analysis revealed altered biological processes related to immune regulation, humoral immune response, proteolysis, and cellular components including plasma lipoprotein particle, extracellular space, and membrane-associated structures. KEGG pathway analysis emphasized enrichment of pathways linked to complement and coagulation cascades, platelet activation, focal adhesion, endocytosis, and inflammation. Together, these data illustrate the potential of LC-MS/MS to examine the role of inflammation and arterial wall cells in shaping the proteome of EVs and plasma in health and disease.

## Introduction

Cardiovascular diseases (CVDs) are the leading cause of mortality in both developed and developing countries, arising from a complex interplay of genetic and environmental factors, with atherosclerosis being the underlying cause in many cases [1]. The pathological progression of atherosclerosis involves endothelial dysfunction, chronic inflammation, lipid accumulation, vascular remodelling, and formation of plaques in the artery wall [2,3]. Acute complications, including myocardial infarction and ischaemic stroke, are primarily triggered by thrombosis and blood clot formation [2,3]. These thrombotic events are most often caused by the mechanical disruption of vulnerable atherosclerotic plaques [4,5]. Early identification of such plaques is critical for clinical intervention, enabling treatment before the onset of acute events [6]. A growing body of evidence links atherosclerosis to increased and chronic inflammation [7–10], with modulation of inflammation and neutrophil numbers showing positive health benefits in large scale clinical trials (CANTOS [11] and COLCOT [12]). The stability of atherosclerotic plaques, and particularly the propensity of these to undergo rupture, a major cause of thrombus formation and arterial occlusion, and therefore occurrence of a stroke or myocardial infarction, has been linked to changes to the proteins that comprise, and provide structural integrity to, the fibrous cap that overlies the necrotic core [13]. Denudation and leakiness of the endothelial cell barrier is also strongly linked to adverse CVD events [14,15]. These processes may result in enhanced leakage of materials, including proteins and fragments derived from these, into the bloodstream, and these might aid early detection and risk stratification of patients [16]. Proteins from both healthy and the diseased artery wall may also appear in the circulation as a result of the release of extracellular vesicles (EVs).

EVs are heterogeneous, membrane-bound particles that include microvesicles, exosomes, and apoptotic bodies. These particles can carry a wide range of biological molecules, such as proteins, RNA, DNA, and lipids [17]. These can include materials specific to the cells and tissue from which they are released, with some of these being bioactive molecules (reviewed [17,18]). These encapsulated biomolecules can therefore reflect the physiological state of donor cells, and influence cells (recipient cells) which are exposed to them through intercellular communication [17,19]. The pivotal role of EVs in cell–cell communication underscores their potential relevance in the pathogenesis of atherosclerosis [20]. EVs are secreted by various cell types involved in cardiovascular health, including endothelial cells, smooth muscle cells, and immune cells [20,21] and may act as reporters of biochemical changes that occur in diseased tissue such as the inflamed artery wall in atherosclerosis. Alterations to the molecular cargo of EVs may reflect CVD status and may provide readily accessible insights into the progression of atherosclerosis via minimally invasive blood sampling [22]. Building on the reported association between EVs and atherosclerosis, we have developed a workflow for proteomic profiling of unfractionated human plasma-derived EVs, unlike a previous study that used plasma fractionated to remove lipoproteins [23]. This workflow includes EV isolation via centrifugation, automated protein digestion, and mass spectrometry-based proteomics analysis, using approaches that build on these previous reports [24]. In the study reported here, EVs from 59 patients with well documented impaired cardiovascular health were compared with 56 healthy controls to identify potential differentially expressed proteins in the isolated EVs. The EV data were also compared to that arising from direct analysis of the plasma from which the EVs were obtained. Enrichment analyses were performed to investigate the biological relevance of the detected proteins and their potential roles in CVD pathology.

## Materials and methods

### Subject recruitment and ethics

Blood samples were collected from subjects enrolled as part of the BIOCLAIMS cohort (606 men and 704 women, aged 18–85 years) of the EU Framework 7 BIOCLAIMS research project recruited at the Medical University and University of Graz, Austria, between May 2011 and November 2014. All study subjects underwent anthropometric, clinical, and biochemical assessment. The two groups (impaired vascular status and healthy subjects) investigated in the present study are part of the BIOCLAIMS Integration (BIG) Study (https://cordis.europa.eu/project/id/244995/reporting/de, retrieved August 31, 2025).

The study was conducted in accordance with the Declaration of Helsinki. The study protocol was approved by the Ethics Committees of the Medical University and University of Graz, Austria [Ethics protocols: University of Graz (EK 23-306 ex 10/11; approval date: May 20, 2011)] and the University of Graz (GZ.39/24/63 ex 2010/11, approval date: May 30, 2011), and a Sample Transfer Agreement was signed on October 3, 2023. Investigation of the study subjects and collection of samples from the subjects was undertaken after written informed consent had been obtained.

### Subject groups

Two groups of the BIOCLAIMS Integration (BIG) study, which was conceived to include four contrasting groups of the entire BIOCLAIMS Cohort, were examined in the present study – a ‘healthy control’ group and a group identified as having impaired vascular health (https://cordis.europa.eu/project/id/244995/reporting/de). The inclusion criteria for the healthy control group, which comprised 56 subjects, were as follows: besides not taking any medications, body mass index (BMI) 18.5–29.9 kg m^−2^, carotid artery intimal media thickness (CIMT) ≤75th percentile for both the left and right sides, homeostatic model assessment of insulin resistance (HOMA-IR) index ≤2.5 mU L^−1^ mM, HbA1c <38 mmol mol^−1^ (with either HOMA-IR or HbA1c allowed above this threshold, but not both), estimated glomerular filtrate rate (eGFR) >60 ml min^−1^/1.73 m^2^, and all clinical chemistry values within the normal range ± 10% [25]. The clinical chemistry test data included: red and white blood cell counts, haemoglobin, hematocrit, thrombocyte count, serum electrolyte, creatinine, urea, uric acid and cystatin C concentrations, γ-glutamyltranspeptidase, cholinesterase, aspartate aminotransferase, alanine aminotransferase activity, pancreatic amylase and lipase activity, serum fasting glucose, C-reactive protein, total cholesterol, HDL and LDL cholesterol, triglyceride, apolipoprotein-A1 and apolipoprotein-B, total protein, albumin, iron, transferrin and ferritin concentrations, and thyroid gland stimulating hormone activity. The corresponding inclusion criteria for the impaired vascular health group (59 subjects) were as follows: CIMT >75th percentile for age and sex on the left and right sides with eGFR >60 ml min^−1^/1.73 m^2^, HOMA-IR index ≤2.5, and HbA1c <38 mmol mol^−1^ (with either the HOMA-IR or HbA1c values allowed above this threshold but not both). A summary of the characteristics of the two subject groups is provided in Supplementary Figure S1 and Supplementary Table S1.

### Preparation of plasma samples

Peripheral venous blood samples were collected using Vacutainer tubes, coated with EDTA, heparin or none after overnight fasting, and processed immediately after venipuncture. Heparin plasma and serum (for clinical chemistry) and EDTA plasma (for the present study) were obtained by centrifugation (1620 *g*, 10 min), and red blood cells were separated from EDTA whole blood by centrifugation (10000 *g*, 1 min) and washed with NaCl (for HbA1c). Clinical chemistry routine variables were analysed the same day, while the other aliquots were stored at −80°C until analysis after dry ice shipment to the Department of Biomedical Science, University of Copenhagen, Denmark.

### Isolation of extracellular vesicles

Two methods were examined for the isolation of EVs: centrifugation and use of a size-exclusion column as detailed below, together with a combination of these as outlined below.

#### Centrifugation isolation

Plasma samples were first centrifuged at 3000 *g* for 5 min, and the supernatant was transferred to fresh tubes. For each 500 μl plasma sample, an equal volume of PBS was added and mixed. Samples were then centrifuged at 24000 *g* for 30 min, and the supernatant discarded. The pellet was resuspended in 1 ml of PBS and centrifuged again at 24000 *g* for 30 min. The final pellet was retained as the isolated EVs.

#### Size-exclusion column isolation

A qEV1 column (Izon Science Ltd., qEV1 1 ml 70 nm) was connected to an Akta FPLC system. Plasma samples (1 ml) were loaded onto the column and eluted with 27 ml of PBS, as per the manufacturer’s instructions. Elution fractions (optimal size: 0.7 ml) were collected throughout the run.

#### Combined centrifugation and SEC

Plasma samples were first processed by the centrifugation method described above. The EV pellet was subsequently diluted to 1 ml in PBS and then subjected to size-exclusion column (SEC) purification as outlined in the preceding paragraph.

### Characterization of EVs using nanoparticle tracking

EV samples were diluted in PBS at a ratio of 1:200 to 1:1000 prior to measurement. Measurements were performed using a ZetaView PMX instrument (Particle Metrix), and data were analysed using ZetaView software (version 8.05.14 SP7). Particle concentrations were adjusted to achieve 30–100 particles per frame during analysis.

### Characterization of EVs using transmission electron microscopy

Formvar/carbon-coated nickel grids (Ted Pella, Inc.) were glow-discharged and incubated with EV samples for 15 min. Samples were sequentially fixed with 2% paraformaldehyde and 2.5% glutaraldehyde, then negatively stained with 2% uranyl acetate. Grids were examined using a CM100 transmission electron microscope (Philips).

### Characterization of EV proteins using liquid chromatography-mass spectrometry (LC-MS/MS)

EVs were lysed in 1% SDS with 10 mM tris(2-carboxyethyl)phosphine (TCEP) and 40 mM 2-chloroacetamide (CAA) to reduce disulfides and subsequently alkylate thiols (70°C, 15 min). Protein concentrations were determined by tryptophan fluorescence spectroscopy using λ_ex/em_ 295/350 nm, and a tryptophan frequency of 1.3% in protein sequences. Aliquots of protein extracts corresponding to 4 μg of protein were transferred to fresh low-binding plates using an Opentron OT2 liquid handling system and digested following an adapted and automated protein aggregation capture (PAC) protocol [26]. Briefly, 40 μg of magnetic beads were added to the samples, followed by acetonitrile [ACN, 70% (v/v) in water]. After mixing, the solution was incubated for 20 min at 21°C. The plates were then placed in a magnetic rack, allowing the beads to separate for 1 min before the supernatant was removed by aspiration. The beads were then washed three times (without release from the magnet) with 100% ACN, and once with 70% (v/v) ethanol in water. The plates were then removed from the magnetic rack, and the beads resuspended in 100 mM triethylammonium bicarbonate (TEAB, pH 8.5) buffer, followed by addition of trypsin (1:50 final ratio of enzyme to protein) and digestion overnight at 37°C. The plates were then placed back in the magnetic rack, and the beads allowed to separate for 1 min. The peptide containing solution was then removed and acidified using trifluoroacetic acid [TFA; 0.5% (v/v) final concentration in water], and stored at −80°C until analysis by LC-MS/MS.

Peptides were separated and analysed using a Dionex Ultimate RSLCnano system equipped with an Aurora C18 column (15 cm × 75 μm, 1.6 μm particle size, IonOpticks) coupled to a Bruker timsTOF Pro mass spectrometer. Peptides were loaded onto a cartridge trapping column containing a 0.17 μl media bed (EXP2 from Optimize Technologies) packed with 10 μm diameter, 100 Å pore PLRP-S beads (Agilent) as described by Kreimer et al. [27] and eluted using a binary gradient [4–25% mobile phase B in 14 min followed by washing and equilibration of the analytical column; mobile phase A: 0.1% (v/v) formic acid (FA) in MS-grade water, mobile phase B: 0.1% (v/v) FA in ACN]. Both trap and analytical columns were maintained at 35°C using an integrated column oven. MS data were acquired using data-independent acquisition (DIA) in parallel accumulation-serial fragmentation (PASEF) mode (DIA-PASEF) [28].

### LC-MS/MS proteomic analysis of plasma

Plasma samples (30 μl) were transferred to fresh low-binding plates, diluted with 1770 μl of 25 mM TEAB (pH 8.5) buffer containing 0.05% *N*-dodecyl-β-d-maltoside (DDM) and incubated at 70°C for 20 min. Diluted and denatured plasma (30 μl) was then transferred to fresh low-binding plates, followed by addition of 40 μl of 50 mM TEAB (pH 8.5) and 30 μl of trypsin solution (0.02 μg/μl). Trypsin digestion was then allowed to proceed for 12 h followed by addition of TCEP and CAA (10 mM and 40 mM final concentration, respectively) and incubation for a further hour. The reactions were then quenched by adding 500 μl 0.5% (v/v) TFA in water and 500 μl MS-grade water, and the samples were stored at −80°C until analysed by LC-MS/MS.

Peptides were separated and analysed using the Dionex Ultimate RSLCnano system and Aurora column described above, except that the peptides were eluted using a shorter gradient [4–25% mobile phase B in 12 min followed by washing and equilibration of the analytical column; mobile phase A: 0.1% (v/v) FA in MS-grade water, mobile phase B: 0.1% (v/v) FA in ACN] with the column oven maintained at 55°C.

### LC-MS/MS data analysis

All data analyses were performed in the R statistical environment. Differential expression analysis was conducted using the *limma* [29] and Msqrob2 packages [30]. Models were fitted with disease status as the main factor, and empirical Bayes moderation was applied to stabilize variance estimates. For biological sex-dependent analyses, samples were stratified by sex and differential analysis was repeated within each subgroup. Statistical significance was defined using an adjusted *P*-value of <0.05 using the Benjamini–Hochberg method [31] to compensate for multiple comparisons and control the false discovery rate (FDR). To assess age-associated differences in protein abundance across disease status, a linear mixed-effects model was implemented using Msqrob2, with fixed effects for age, disease status, and their interaction, and patient ID as a random effect. Significant differentially abundant proteins were subjected to overrepresentation analysis (ORA) using the clusterProfiler [32] and Disease Ontology Semantic and Enrichment (DOSE) packages [33], with a Benjamini–Hochberg adjusted *P*-value of <0.01 set as the cut-off. Gene Ontology (GO) terms for biological process, molecular function, and cellular component were assessed separately. Cell type enrichment analysis was performed using WebCSEA (https://bioinfo.uth.edu/webcsea) and the corresponding R wrappers. Matrisome proteins were annotated based on the MatrisomeDB database [34], and classified into core matrisome, matrisome-associated, or non-matrisome categories.

### Biomarker measurements using ELISA

Plasma concentrations of multiple biomarkers were established using enzyme-linked immunosorbent assays (ELISAs). Biomarkers for collagen type III degradation, collagen type VI degradation, collagen type IV degradation, and collagen type IV turnover were measured using the nordicC3M^™^, nordicC6M^™^, nordicC4M^™^, and nordicPRO-C4^™^ assays, respectively. Other biomarkers included in the panel were nordicPRO-C6^™^, nordicCAN^™^, and nordicC1SIG^™^, which quantify endotrophin from collagen type VI, canstatin derived from collagen type IV and a novel signalling peptide derived from collagen type I, respectively. All biomarkers were measured as described previously [35–39], according to the manufacturers’ guidelines (Nordic Bioscience A/S, Denmark). The manufacturer’s quality control samples were included in every assay to confirm that standard curves passed all criteria. The coefficient of variance percentage (CV%) for the plasma samples did not exceed 10% (nordicC3M^™^, nordicC4M^™^, and nordicPRO-C4^™^), 20% (nordicPRO-C6^™^, nordicC6M^™^) or 25% (nordicCAN^™^, nordicC1SIG^™^) based on the biomarkers’ level of technical validation and internal quality control criteria.

### Comparison with published EV proteomics datasets

For comparison with previously published EV proteomics studies, protein lists were extracted from the Supplementary Materials of two previous studies [40,41]. Protein identifiers were harmonized using gene symbols where possible and compared with the protein identifications from the current EV dataset. The overlap between datasets was then assessed. In addition, proteins highlighted by the original studies [40,41] as important or of particular interest were specifically examined in the current dataset. This analysis was intended as a contextual comparison with published EV proteomics data, rather than as validation in an independent cohort.

### Statistical analysis

All statistical analyses were performed using the R package (version 4.4.3). Proteomics data processing, including normalization, differential abundance analysis, and enrichment testing, was conducted as described above (see LC-MS/MS data analysis). Statistical significance in all tests was defined using a FDR threshold of 0.05, corrected by the Benjamini–Hochberg method [31] unless otherwise noted. For the ELISA-derived biomarker measurements, data were imported into R and visualized using the ggplot2 package. Statistical comparisons between groups were performed using Wilcoxon rank-sum tests using the ggpubr package.

## Results

### Isolation and identification of EVs from human plasma

**Figure 1:**
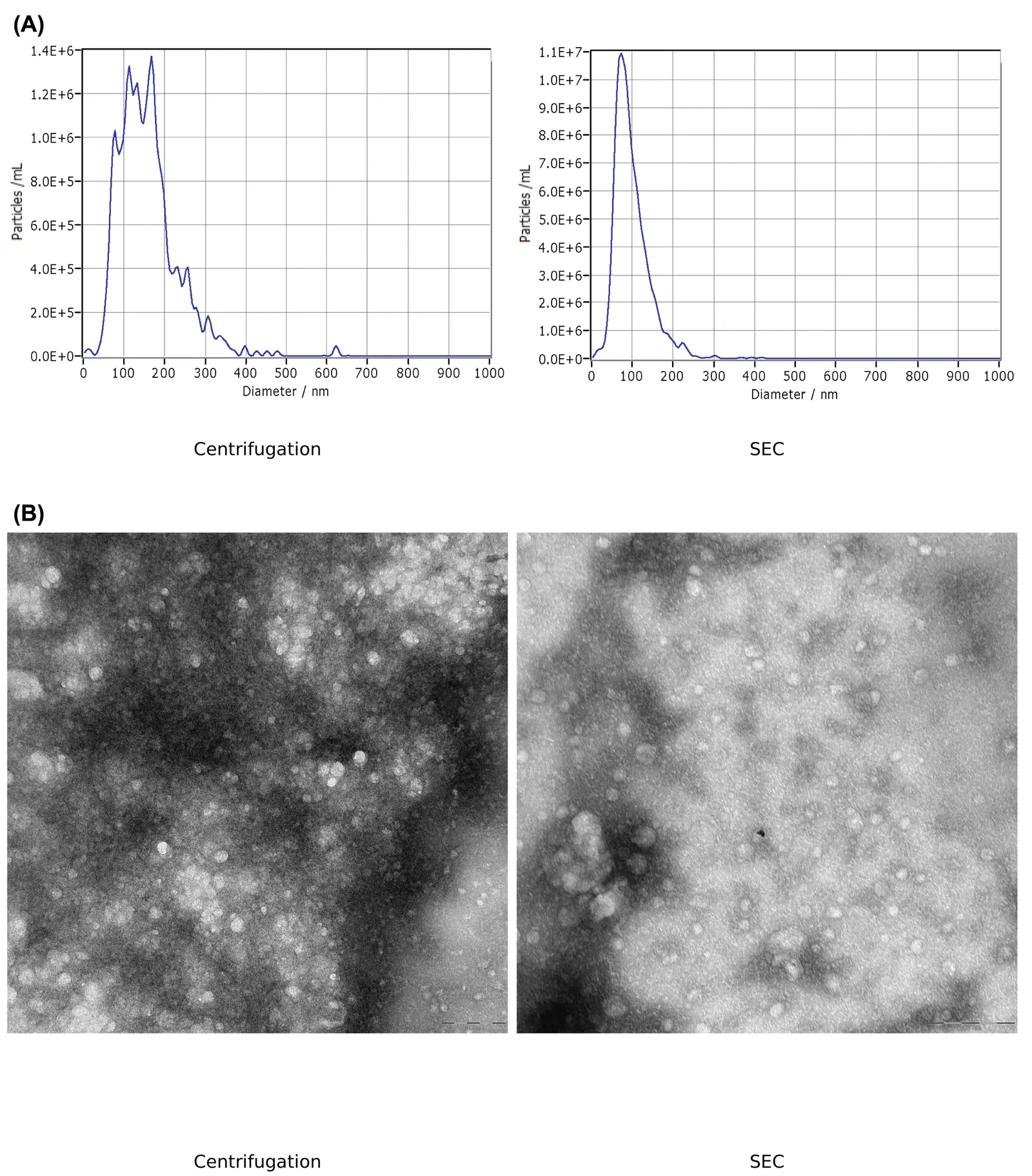
Isolation and characterization of EVs (**A**) NTA of EVs isolated by centrifugation (right) and SEC (left). EV samples were diluted in PBS at a ratio of 1:200 to 1:1000 prior to measurement and particle concentrations were adjusted to achieve 30–100 particles per frame during analysis. For further details see Materials and methods. (**B**) TEM images of EVs isolated by centrifugation (right) and SEC (left). EVs were adsorbed onto glow-discharged Formvar/carbon-coated nickel grids, fixed with paraformaldehyde and glutaraldehyde, negatively stained with 2% uranyl acetate, and imaged using a Philips CM100 TEM. For further details see Materials and methods. Scale bars in (B) are 200 nm.

To establish an effective workflow for EV isolation in plasma-based proteomics, we initially combined high-speed centrifugation (24000 *g*) with SEC (IZON column) to maximize purification of materials from standard (healthy subject) plasma. Although this two-step method generated cleaner EV-enriched preparations with reduced soluble plasma protein contamination, the resulting particle concentration was too low for reliable and reproducible LC-MS detection of proteins, though this approach has been reported to be successful with other samples [42]. We therefore compared centrifugation and SEC individually, using the standard plasma sample, to evaluate both their separation efficiency and practical utility. Nanoparticle Tracking Analysis (NTA) was performed to assess the particle size distribution of EVs isolated by each method. SEC yielded a narrowly distributed particle population, while centrifugation resulted in a broader peak centred at a similar size range (Figure 1A). Transmission Electron Microscopy (TEM) supported the presence of vesicle-like particles in the samples generated using both methods, indicating successful EV isolation by both approaches (Figure 1B).

**Figure 2:**
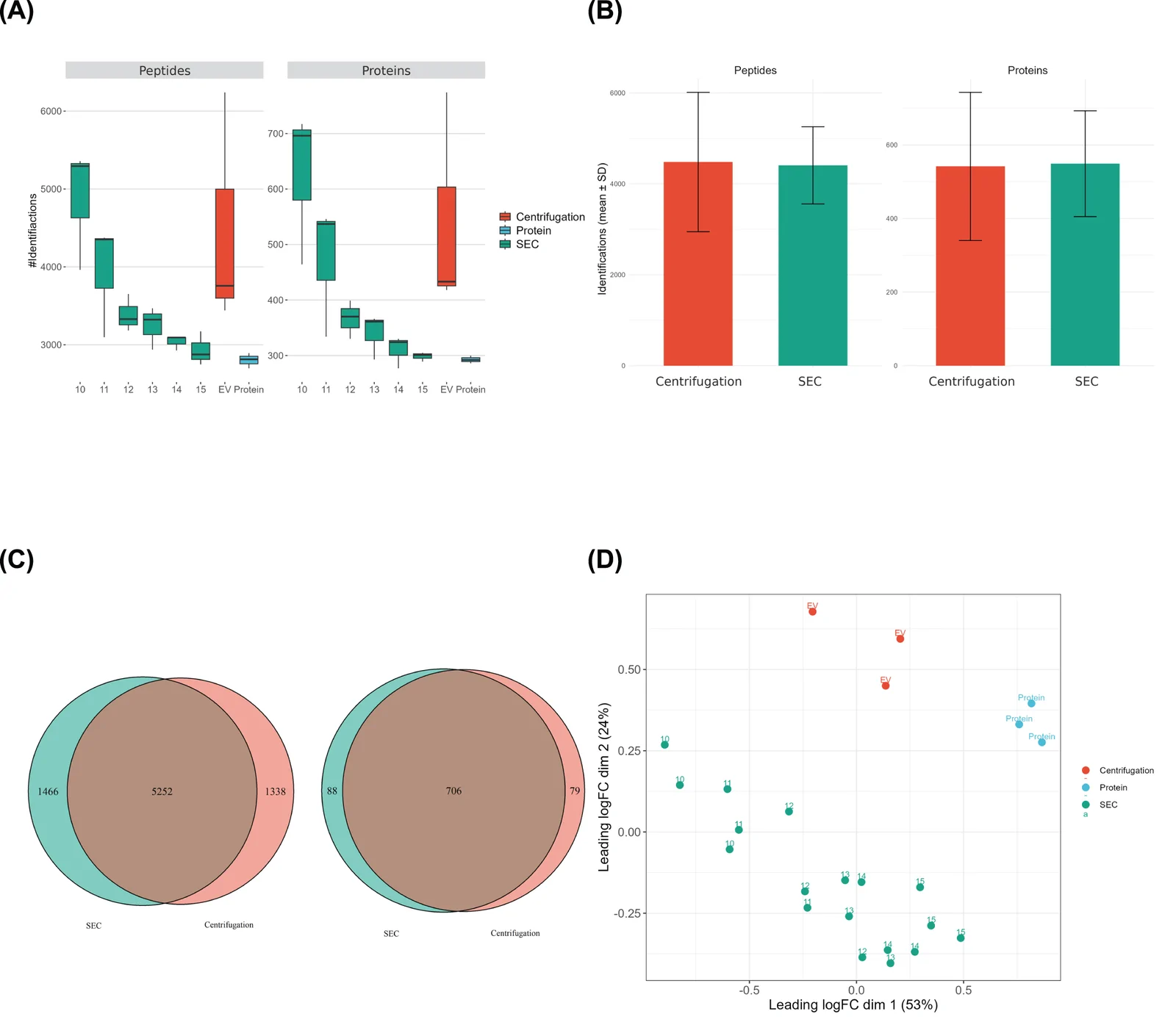
Peptides and proteins identified from EVs obtained by centrifugation (‘EV’), SEC-enriched EV fractions (fractions 10–15), and SEC-derived protein fractions (fraction 22) (**A**) Boxplots showing the number of identified peptides (left) and proteins (right) in EVs obtained by the different approaches. (**B**) Barplots showing the number of identified peptides (left) and proteins (right) in EVs obtained by centrifugation and SEC (fractions 10 and 11: as recommended by the column manufacturer). (**C**) Venn plots showing the overlap of identified proteins (left) and peptides (right) in EVs obtained from centrifugation (red) and SEC (green; fractions 10 and 11). (**D**) MDS plot of the proteomic profiles obtained from the different EV preparation methods and fractions (centrifugation, red; SEC, turquoise; Protein fraction, blue) with the numbering indicating the different methods and the fractions indicated in panel (**A**).

To evaluate protein yield and detectability, EVs obtained from both methods were subjected to LC-MS/MS (proteomic) analysis. As shown in Figure 2A, the number of peptide and protein identifications detected from these samples varied across the different SEC fractions, with fractions 10 and 11 (as recommended by the manufacturer) yielding the highest number of peptides and proteins associated with the EV preparations. In contrast, the last SEC fraction (fraction 22; labelled ‘protein’) contained predominantly soluble plasma proteins, consistent with protein-enriched fractions rather than EVs. Samples obtained via the centrifugation protocol yielded a comparable number of identified proteins to the SEC-enriched EV fractions (Figure 2B). In addition, there was a high degree of commonality in both the protein and peptide identification detected from both methods (Figure 2C). Multidimensional scaling (MDS) analysis did not reveal any distinct clustering differences between EVs prepared using centrifugation versus SEC (Figure 2D) suggesting that there is no inherent bias in the identifications obtained between the two approaches.

**Figure 3:**
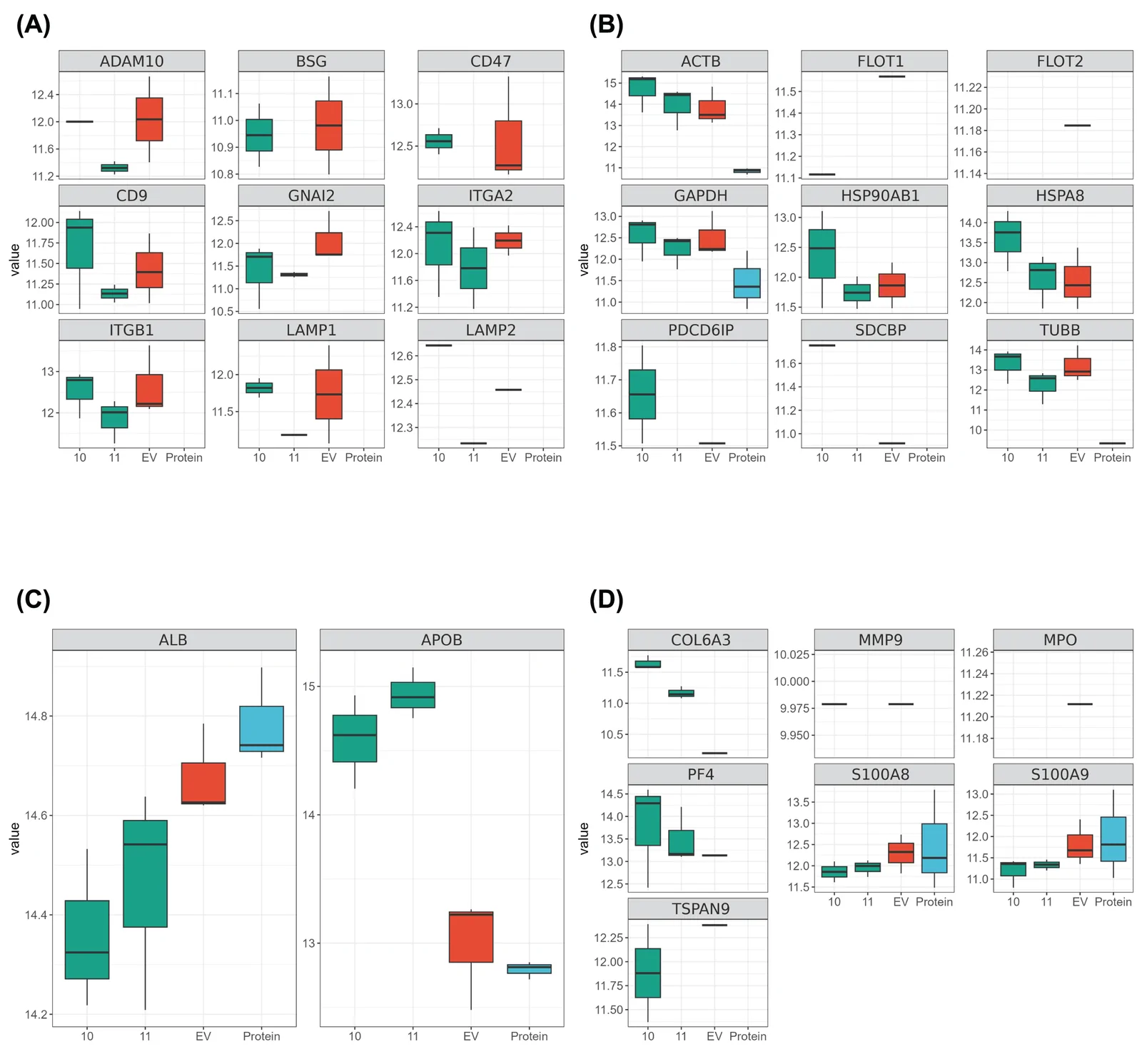
Distribution pattern of proteins across different fractions obtained from both the centrifugation (‘EV’, red colour) and SEC methods of EV preparation (fractions 10-11; turquoise colour) and the protein-enriched sample from SEC (fraction 22, ‘Protein’, blue colour) (**A**) Distribution profiles for the EV-associated proteins [43]: a disintegrin and metalloproteinase domain-containing protein 10 (ADAM10), basigin (BSG), cluster of differentiation 47 (CD47), cluster of differentiation 9 (CD9), guanine nucleotide-binding protein g(i) subunit α-2 (GNAI2), integrin subunit α2 (ITGA2), integrin subunit β1 (ITGB1), lysosome-associated membrane glycoprotein 1 (LAMP1), and lysosome-associated membrane glycoprotein 2 (LAMP2). (**B**) Distribution profiles for the cytosolic proteins in EVs [43]: actin β (ACTB), flotillin 1 (FLOT1), flotillin 2 (FLOT2), glyceraldehyde-3-phosphate dehydrogenase (GAPDH), heat shock protein 90 α family class A member 1 (HSP90AB1), heat shock protein family A member 8 (HSPA8), programmed cell death 6 interacting protein (PDCD6IP), syntenin-1/syndecan binding protein (SDCBP), tubulin β class I (TUBB). (**C**) Distribution profiles for the abundant plasma proteins human serum albumin (ALB) and apolipoprotein B100 (APOB). (**D**) Distribution profiles for potential disease-associated proteins: collagen type VI α3 chain (COL6A3), matrix metallopeptidase 9 (MMP9), myeloperoxidase (MPO), platelet factor 4 (PF4), s100 calcium binding protein a8 (S100A8), s100 calcium binding protein a9 (S100A9), and tetraspanin 9 (TSPAN9).

A more detailed analysis was carried out on the distribution of a number of proteins identified across the different SEC fractions, and by the centrifugation approach. Analysis of the proteins detected from these two methods showed the presence of multiple well-established markers of EVs in these fractions, including ADAM10, BSG, CD47, CD9, GNAI2, ITGA2, ITGB1, LAMP1, LAMP2, ACTB, FLOT1, FLOT2, GAPDH, HSP90AB1, HSPA8, PDCD6IP, SDCBP, and TUBB as reported in the MISEV2023 guidelines [43]. A total list of the protein identifications detected by each method is presented in Supplementary Table S2. The distribution of proteins is presented in Figure 3, with a number of these showing a broad distribution across the fractions obtained from the SEC (fractions 10 and 11). As expected, SEC showed a superior ability to reduce the level of plasma proteins such as albumin (ALB) and apolipoprotein B100 (APOB), indicating its greater purification efficiency (Figure 3C). In contrast, a number of additional proteins showed a differential distribution across the SEC fractions consistent with the cargo of the EVs also containing inflammation and ECM proteins.

Together these data indicate that both sample preparation methods (centrifugation and SEC) yield EVs with comparable proteomic profiles, albeit with less selectivity in the case of centrifugation. The latter is however considerably less complex, less time-consuming and more suitable for the preparation of large numbers of samples (i.e. has a higher throughput). In the light of this similarity in performance, centrifugation was chosen as a more practical approach for EV isolation for studies on the clinical cohorts under study.

### Proteomic analysis of EVs from subjects with impaired vascular health versus controls

Based on the workflow outlined above, the samples from the BIOCLAIMS project were processed, using the centrifugation method, to explore potential differences in the protein cargo of the EVs isolated from the subjects with impaired vascular health versus healthy controls. After EV isolation and automated processing (see Materials and methods), the samples were randomly distributed and analysed as technical triplicates, across multiple plates to minimize batch effects during LC-MS/MS analysis.

**Figure 4:**
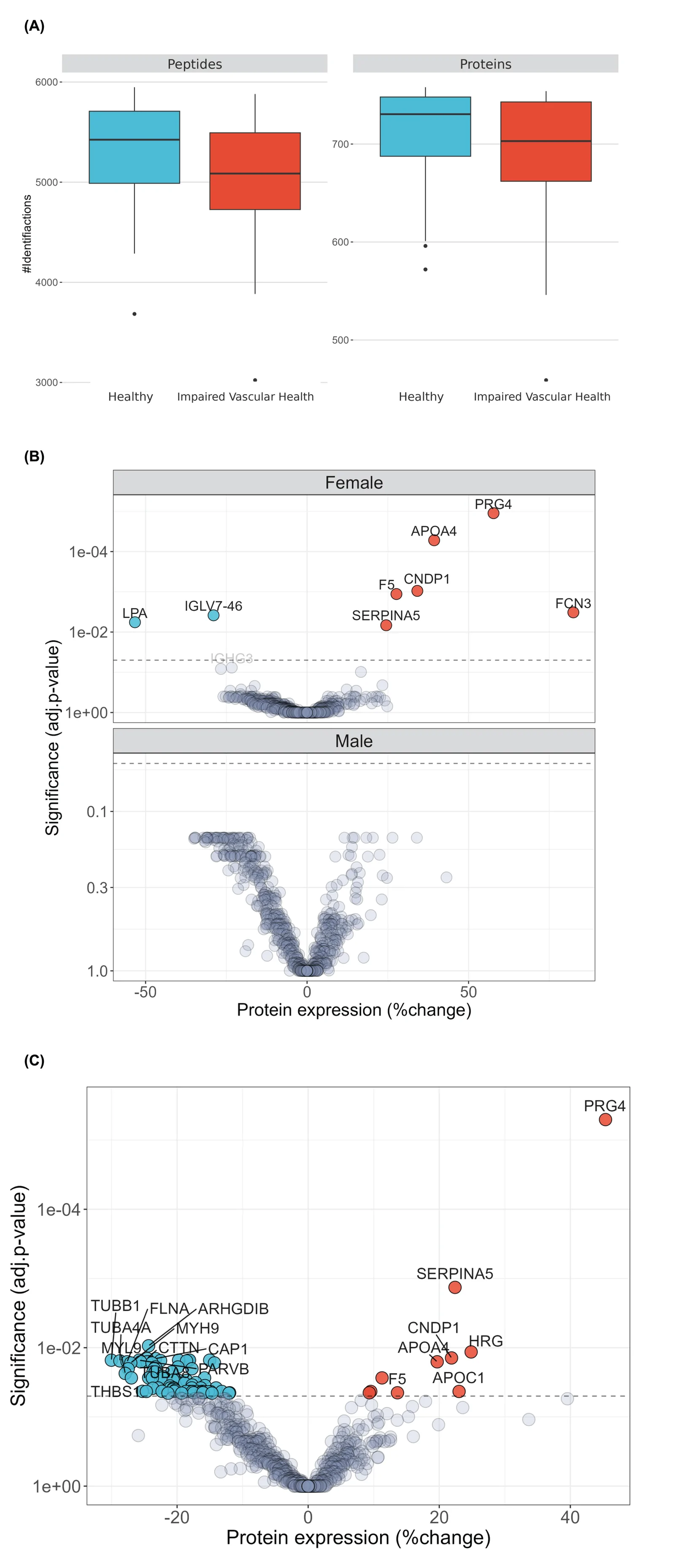
Proteomic analysis of EVs obtained from plasma from the ‘healthy’ and ‘impaired vascular health’ groups (**A**) Number of peptides and proteins identified by LC-MS/MS of EVs obtained from centrifugation of plasma samples from the ‘healthy’ and ‘impaired vascular health’ groups. (**B**) Volcano plot of differences in protein abundance (% change in expression) in EVs from the impaired vascular health and healthy groups. (**C**) Volcano plot of differences in protein abundance (% change in expression) in EVs from the impaired vascular health and healthy groups segregated on the basis on biological sex (top panel: female; bottom panel: male). For both (**B,C**), the *x*-axis represents percentage change in protein expression, and the *y*-axis shows the adjusted *P*-values (−log10 scale). The grey symbols indicate proteins that were not significantly different (adjusted *P*>0.05, Benjamini–Hochberg method) in concentration between the two groups. Red symbols indicate proteins that were detected as significantly more abundant (adjusted *P*-value < 0.05) in the impaired vascular health groups, and blue symbols those that were more abundant in the healthy group.

This approach resulted in the identification of 7228 unique peptides and 763 proteins in total across all the EV samples (Figure 4A). An average of 687 proteins (range: 459–754) were identified per sample in the impaired vascular health group, and 712 (range: 572–758) proteins in the healthy group. Differential abundance analysis was performed on the total pool of proteins (i.e. 763) using the *Limma* and Msqrob2 packages in R. A FDR-adjusted Benjamini–Hochberg method [31] was used to identify proteins that were significant differentially abundant, at the *P* < 0.05 level, between the healthy and impaired vascular status groups. This resulted in the identification of 87 proteins as indicated in the Volcano plot shown in (Figure 4B), with the complete list of proteins provided in Supplementary Table S2.

Subgroup analyses were also performed to identify any biological sex differences, with this carried out for male and female participants separately. No statistically significant protein differences were observed in the male subgroup between the healthy and impaired vascular health groups, whereas samples from the female subjects exhibited 8 differentially expressed proteins, with 6 being over-abundant in the impaired vascular health group, and 2 in the healthy controls (Figure 4C and Supplementary Table S3).

**Figure 5:**
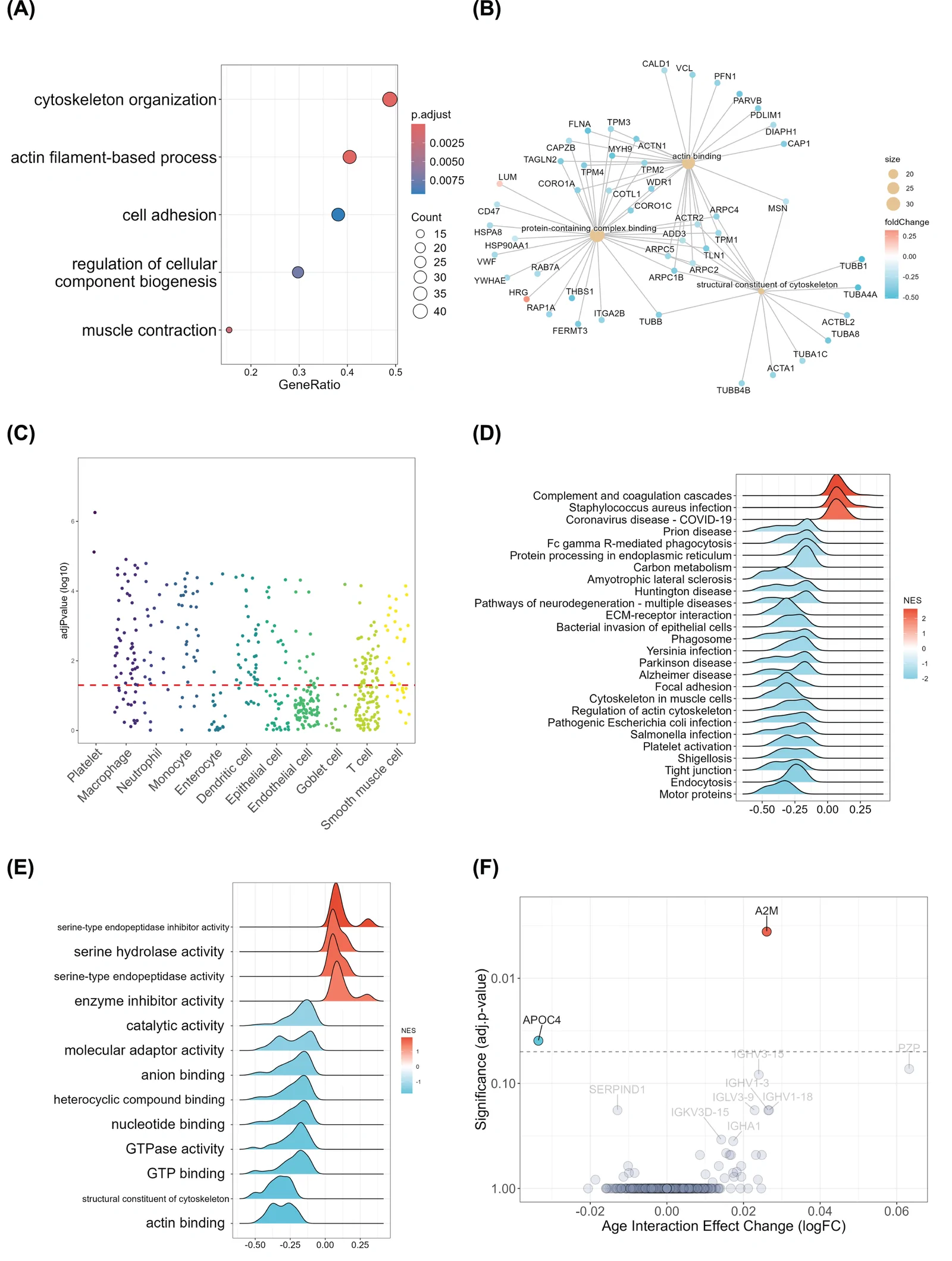
Cluster analysis of the detected proteins across both biological sexes and between the impaired vascular health and healthy subject groups (**A**) ORA of gene ontology biological processes. Significantly enriched processes are shown for proteins detected with differential expression levels between the two groups. The size of the dots represents the number of proteins involved (Count), and colour indicates the Benjamini–Hochberg adjusted *P*-value (p.adjust). The horizontal axis (GeneRatio) refers to the proportion of input proteins annotated to each GO term. (**B**) ORA gene ontology molecular function. Nodes represent enriched GO terms (large labels) and associated proteins (small circles), coloured by fold change. Node size reflects the number of proteins linked to each term. Terms are from the molecular function (MF) category. (**C**) Cell type enrichment of differentially expressed proteins, plotted with adjusted *P*-value on the vertical axis and cell type on the horizontal axis. Proteins were analysed following the WebCSEA workflow (https://bioinfo.uth.edu/webcsea). Each dot represents the adjusted *P*-value (log10 scale) of enrichment for a given cell type. The red dashed line indicates the significance threshold. (**D**) GSEA using KEGG pathways. Each ridge plot shows the distribution of enrichment scores for a given pathway or term. The normalized enrichment score (NES) is indicated by the colour scale (red = positive NES, blue = negative NES), reflecting whether the gene set is enriched in up-regulated or down-regulated proteins. (**E**) GSEA using MF terms. For further details see Materials and methods and text. (**F**) Association of differences in protein abundance with subject age for the healthy (blue symbol) and impaired vascular health (red symbol) groups for the 87 significantly different proteins. Grey symbols indicate non-significant data. The *x*-axis represents the interaction effect size (log fold change) between age and vascular health status, derived from a linear mixed-effects model. Positive values (right side) indicate proteins with stronger age-related increase in expression in the impaired vascular health group, while negative values (left side) indicate stronger age effects in the healthy group.

The proteins detected across both biological sexes were subjected to cluster enrichment analysis. In overrepresentation enrichment analysis for 87 significantly different proteins, the most highly enriched Gene Ontology biological processes included cytoskeletal organization and actin filament-based processes (Figure 5A). In the molecular function category, actin binding and structural constituent of cytoskeleton were identified as key enriched terms (Figure 5B). Cell-type-specific enrichment analysis revealed strong associations with atherosclerosis-related cell types, including platelets, macrophages, and smooth muscle cells (Figure 5C), consistent with the EVs retaining (at least in part) signatures from the originating tissue and cells (in this case the diseased arterial wall and plaques). Gene set enrichment analysis (GSEA) based on all detected proteins, using the Kyoto encyclopedia of genes and genomes (KEGG) pathways (Figure 5D) and biological process (BP) terms, consistently highlighted the central role of the cytoskeleton (Figure 5E).

To explore possible age-dependent differences in protein abundance between the healthy and impaired vascular health groups, an interaction model was fitted using the Msqrob2 package. A linear mixed-effects model was constructed with disease status, age and their interaction as fixed effects, and patient ID as a random effect. Robust and ridge options were enabled to stabilize variance estimates. As shown in Figure 5F, only A2M (for the impaired vascular health group) and APOC4 (healthy subjects) exhibited statistically significant interaction effects with the age of the subjects.

### Proteomic analysis of plasma from subjects with impaired vascular health versus controls

**Figure 6:**
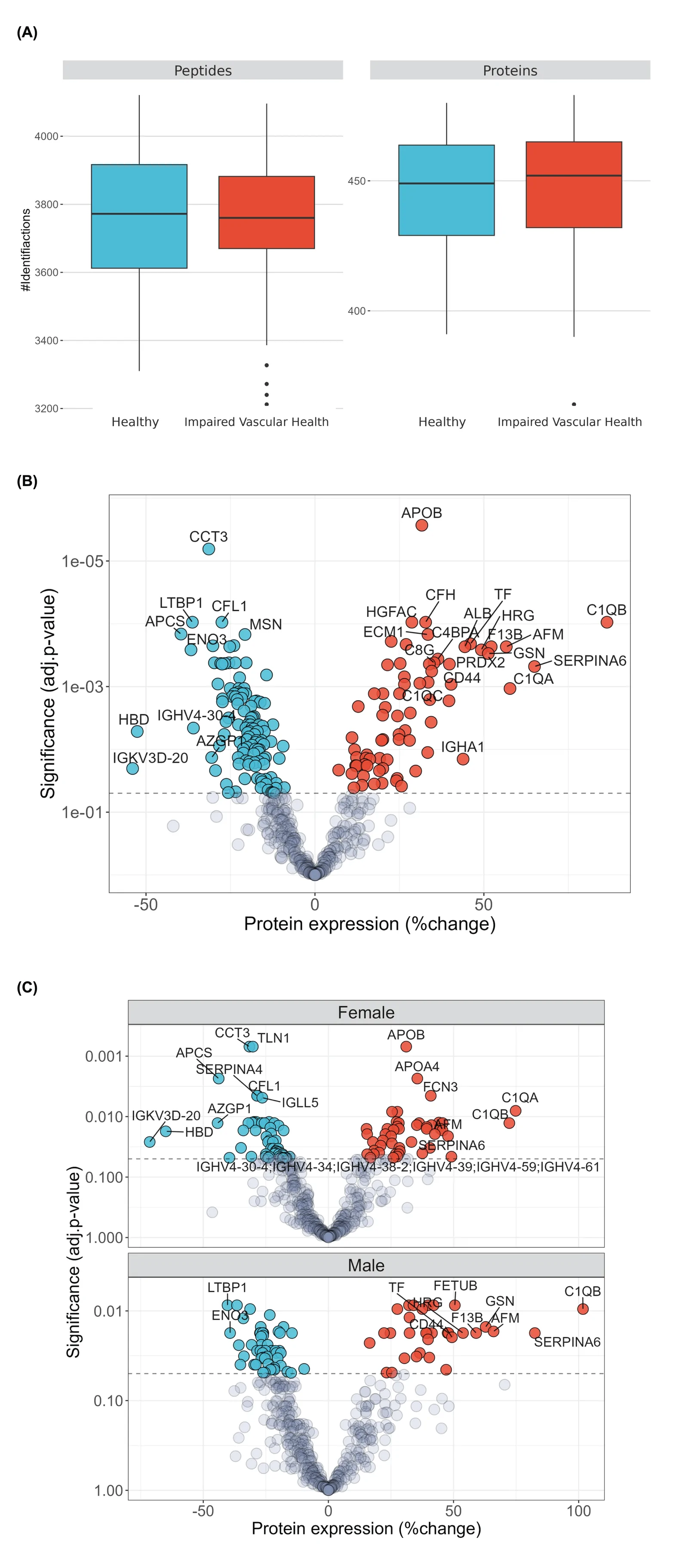
Total peptide and protein identifications in plasma samples from individuals with impaired vascular health and healthy controls (**A**) Boxplots show the number of proteins identified by mass spectrometry in each group. (**B**) Volcano plot of differential plasma protein expression between the healthy and impaired vascular health groups. (**C**) Sex-stratified volcano plots of differential plasma protein expression between healthy and impaired vascular health groups, with the top section providing data for the female subjects and the bottom section the male participants. For both panels (**B,C**), the *x*-axis represents protein expression change (%), and the *y*-axis shows the Benjamini–Hochberg adjusted *P*-value (−log10 scale). Proteins significantly enriched in the healthy group (blue) or impaired vascular health group (red) are highlighted; non-significant proteins are shown in grey. Selected proteins are labelled.

To provide additional data on the subjects and provide context for the above EV data, analyses were also carried out on the non-fractionated plasma from these subject groups, to allow comparison between the plasma and EV data. These analyses employed the same analytical pipeline (see Materials and methods). Following protein extraction, tryptic digestion and LC-MS/MS analysis, 4366 peptides corresponding to 497 unique proteins were identified across all plasma samples (Figure 6A). On average, 447 proteins (range: 364–483) were identified in the impaired vascular health group and 446 (range: 391–480) in the healthy controls, indicating a comparable depth of proteome coverage across conditions.

Differential abundance analysis was conducted using the *Limma* and Msqrob2 R packages, with significance defined with a FDR adjusted *P*-value of <0.05. This resulted in the identification of 188 proteins with significantly altered abundance between the two groups (Figure 6B). A list of these proteins is provided in Supplementary Table S4. Subgroup analysis by biological sex revealed a total of 103 significant differentially expressed proteins in female participants and 84 in male participants (Figure 6C); these proteins are listed in Supplementary Table S5. These data point toward both shared and sex-specific proteomic signatures of vascular impairment.

**Figure 7:**
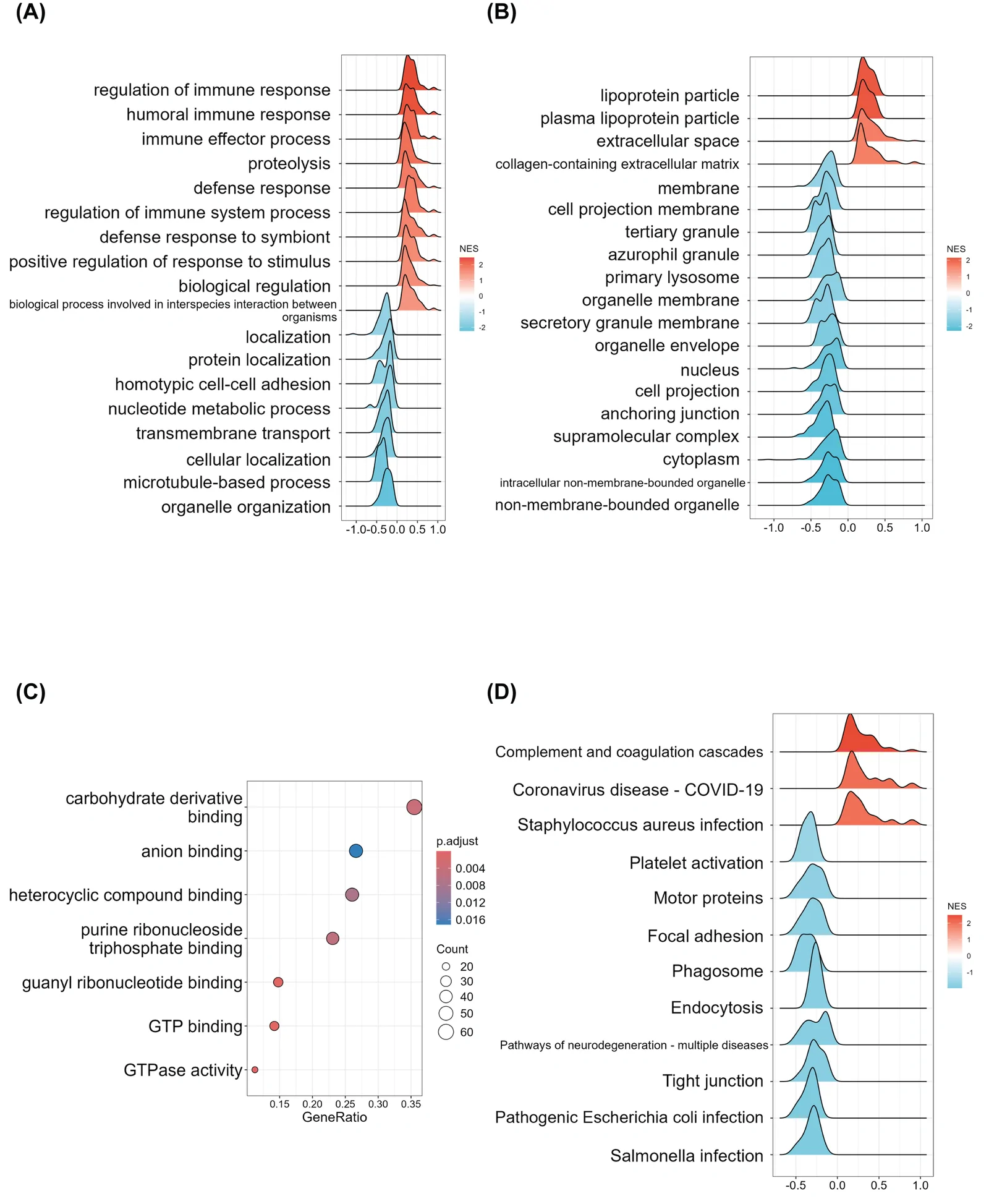
Cluster analysis of detected proteins in plasma between the healthy and impaired vascular health groups Significantly GSEA enriched biological processes are shown in panel (**A**) and cellular component in panel (**B**). Each ridge plot shows the distribution of enrichment scores for a given pathway or term. The NES is indicated by the colour scale (red = positive NES, blue = negative NES), reflecting whether the gene set is enriched in upregulated or downregulated proteins. (**C**) Molecular function enrichment ORA analysis of the differentially expressed plasma proteins. Dot size represents the number of proteins involved (Count), and the colour coding indicates the Benjamini–Hochberg adjusted *P*-value (p.adjust). GeneRatio refers to the proportion of input proteins annotated to each GO. (**D**) GSEA using KEGG pathways.

Gene Set Enrichment Analysis (GSEA) based on all 497 detected proteins revealed biological processes related to immune regulation, humoral immune response, proteolysis, and cellular localization (Figure 7A), as well as cellular components including plasma lipoprotein particle, extracellular space, and various membrane-associated structures (Figure 7B). ORA of the 188 significantly altered proteins, using all 497 proteins as background, highlighted molecular functions such as anion binding, guanyl ribonucleotide binding, GTP binding, and GTPase activity (Figure 7C). In addition, GSEA of KEGG pathways emphasized enrichment of pathways linked to complement and coagulation cascades, platelet activation, focal adhesion, endocytosis, and infection-related processes (Figure 7D). Together, these analyses underscore the interconnected roles of immune regulation and metabolic signalling in shaping the plasma proteome.

These enrichment analyses identified multiple pathways closely linked to the pathophysiology of atherosclerosis, including lipoprotein transport, immune complex circulation and ECM binding. However, a substantial proportion of the enriched terms are related to general plasma functions reflecting the multifaceted nature of the blood proteome. Potential age-dependent differences in protein abundance were also examined between the two groups using a linear mixed-effects model. While some proteins displayed trends suggestive of group-specific age effects, no statistically significant interactions were detected between age and vascular health status for this plasma dataset (Supplementary Figure S2) unlike the EV data.

### Comparison of protein identifications between plasma and EVs from subjects with impaired vascular health versus controls

**Figure 8:**
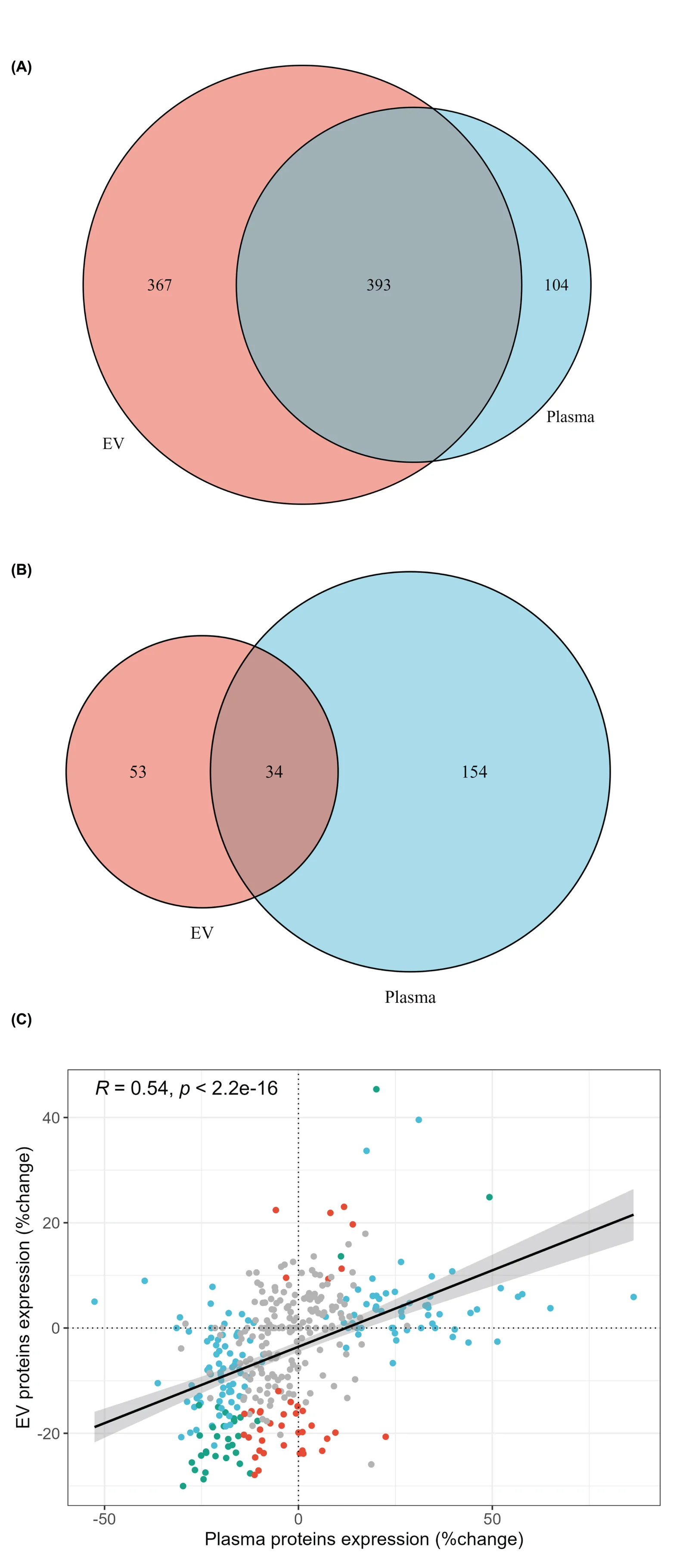
Comparison of data obtained from the plasma and EV proteomic datasets (**A**) Venn diagram indicating the extent of overlap of the proteins identified in plasma (blue) and EV (red) proteomic datasets. A total of 393 proteins were commonly detected in both sample types, while 367 and 104 proteins were exclusively identified in the EV samples and plasma respectively. (**B**) Venn diagram showing the extent of overlap of the differentially expressed proteins between the plasma (blue) and EV (red) samples. 34 proteins were commonly detected in both sample types, while 53 and 154 proteins were exclusively identified in the EVs and plasma respectively. (**C**) Spearman correlation analysis of protein expression changes (%change) for proteins shared between the plasma and EV proteomes. Each point represents a protein detected in both sample types. Colours indicate statistical significance with regard to differential expression: red – EV alone; blue – plasma alone; green – both plasma and EVs; grey – none. The black line shows the linear regression trend together with the 95% confidence interval.

To evaluate the relationship between the detected EV and plasma proteomes, we compared the protein identifications and differential expression patterns between these samples. A total of 393 proteins were shared between the two datasets, while 367 proteins were exclusively detected in EVs, and 104 were unique to plasma (Figure 8A). A list of these shared and distinct proteins is provided in Supplementary Table S6. This indicates that while EVs and plasma exhibit partially overlapping proteomes, each compartment also includes a distinct set of proteins that may reflect distinct aspects of vascular pathophysiology.

Among the 188 plasma and 87 EV proteins identified as significantly different in abundance between the healthy and impaired vascular function groups, 34 were shared (Figure 8B and Supplementary Table S7). Spearman correlation analysis of the protein expression change (% change) for the 393 shared proteins showed a moderate positive correlation (*R:* 0.55, *P* < 2.2 e-16) suggesting a consistent rank order of protein expression changes between the plasma and EVs, indicating a partial concordance of disease-associated proteomic signals (Figure 8C). However, a significant number of the differentially expressed proteins were exclusive to either the plasma (154 proteins) or EV samples (53 proteins), indicating sample-type-specific responses to vascular impairment (Figure 8B).

**Figure 9:**
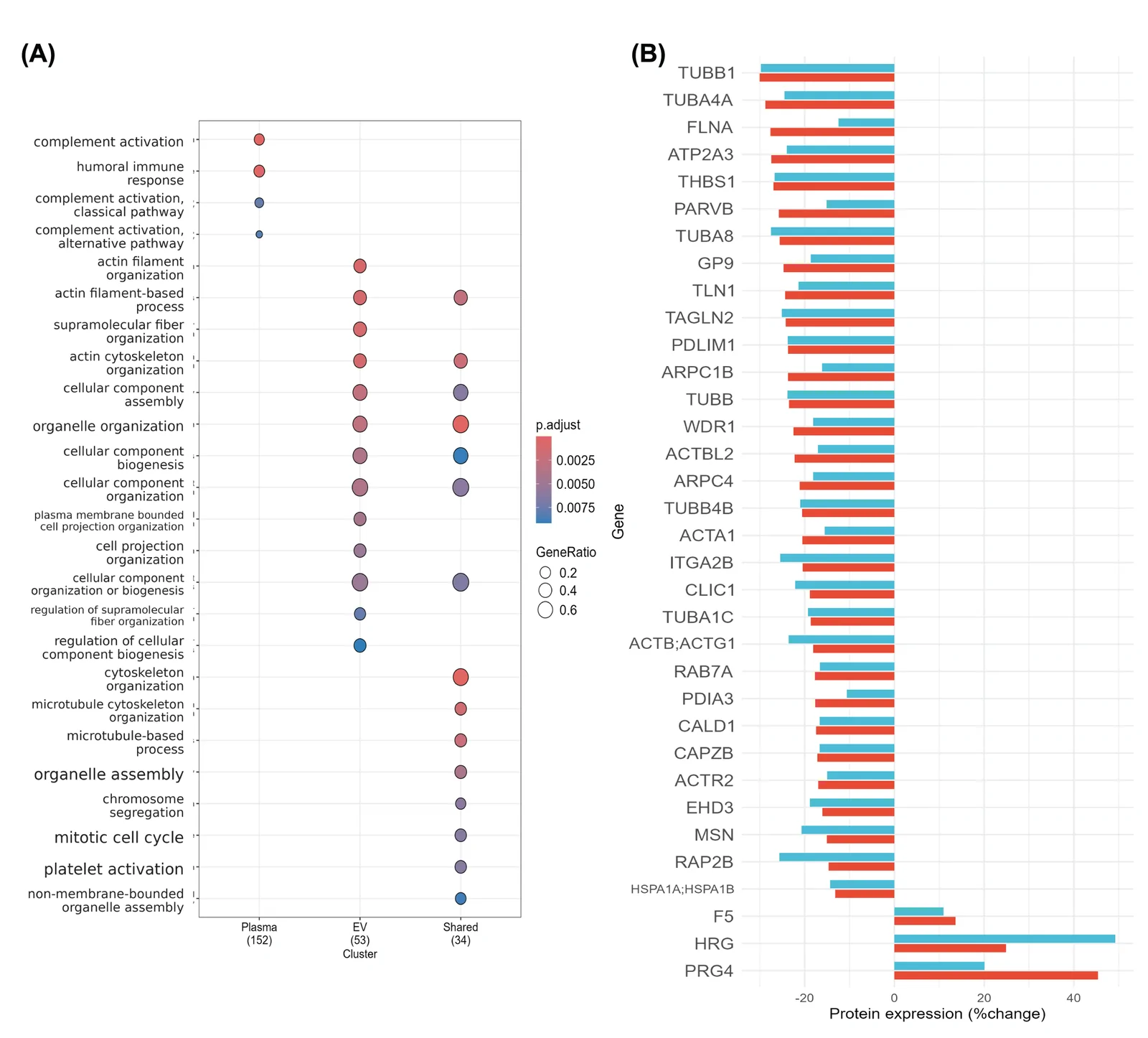
Comparison of GO biological process enrichment analysis among plasma, EV and shared significantly different proteins (**A**) GO enrichment analysis was performed separately for the differentially expressed proteins that were uniquely significant in plasma, in EVs (EV), and shared significant proteins identified in both sample types (Shared). Dot colour represents adjusted *P*-value (p.adjust), and dot size reflects the gene ratio (GeneRatio), indicating the proportion of proteins involved in each term relative to the input list size. (**B**) Comparison of differential expression of significantly regulated proteins shared between plasma and EVs. Each gene is represented by two bars showing the direction and magnitude of the change in EVs (red) and plasma (blue). Positive values indicate upregulation, whereas negative values indicate downregulation.

Gene Ontology (GO) enrichment analysis also revealed both shared and distinct biological processes enriched among the differentially expressed proteins from plasma, EVs and the overlapping subset (Figure 9A). Shared proteins were enriched in immune response, complement activation, and platelet degranulation pathways, consistent with systemic inflammation and vascular stress. Notably, EV-specific enrichments included cytoskeletal organization and actin-binding functions, whereas plasma-specific terms highlighted lipoprotein metabolism and coagulation. A direct comparison of expression trends for the 34 shared differentially expressed proteins showed that most proteins exhibited similar directionality of change in plasma and EVs, although the magnitude often varied (Figure 9B). These results support the notion that EVs can reflect disease-associated systemic proteomic changes found in plasma, while also providing additional cell- and tissue-specific information not readily detectable in whole plasma.

### Measurement of specific extracellular matrix proteins and fragments in EVs and plasma from subjects with impaired vascular health versus controls

To assess the potential of selected ECM-related proteins (and fragments from these) as biomarkers of vascular impairment, plasma levels of several collagen-associated proteins were assessed using both proteomics and ELISA.

**Figure 10:**
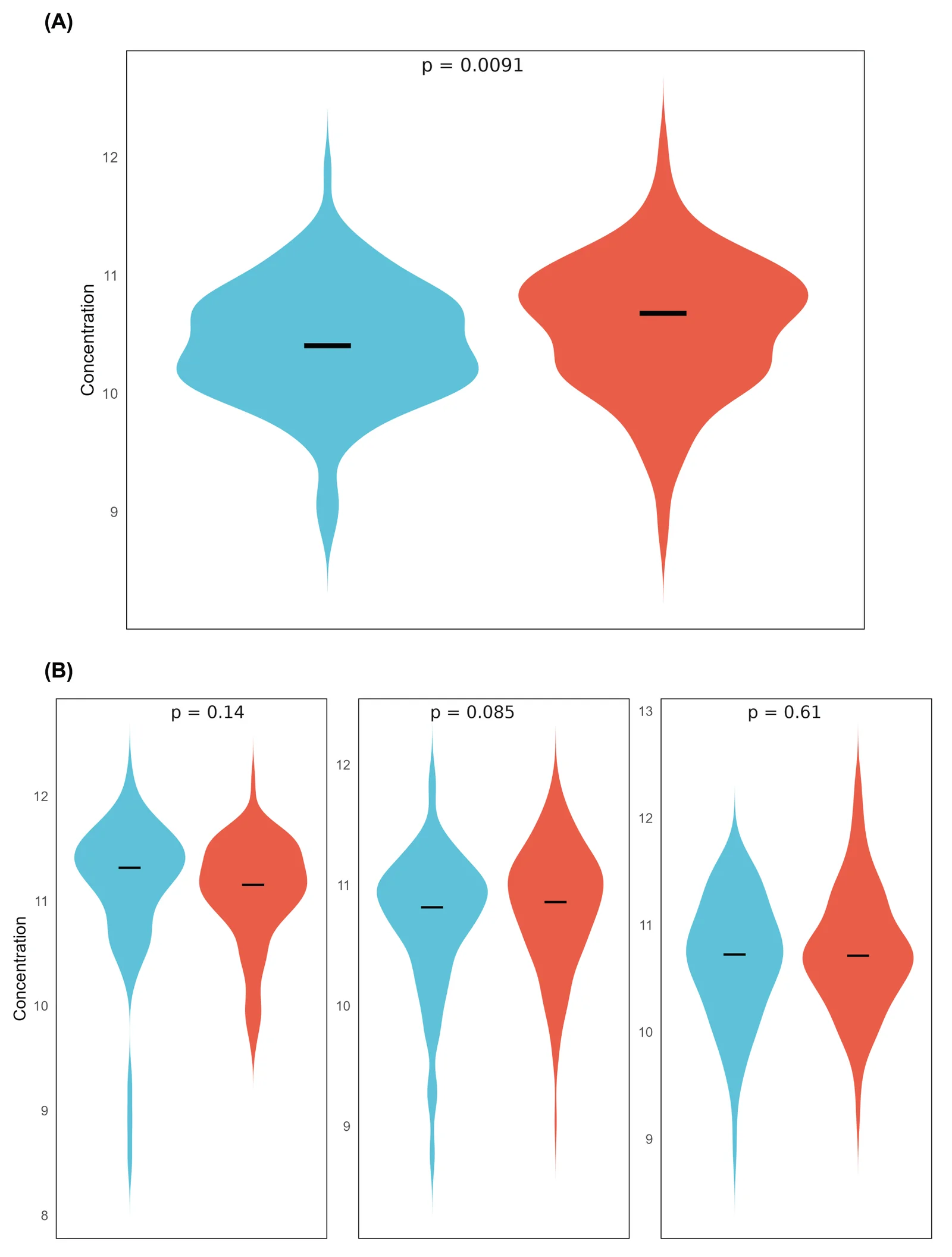
Level of collagen COL6A3 in the EV proteomics data from participants with impaired vascular health (red colour) compared to healthy controls (blue colour) (**A**) Violin plots showing the significantly greater (Wilcoxon rank-sum test, *P*=0.0091) expression level of collagen COL6A3 in the EV proteomics data from participants with impaired vascular health (red colour) when compared to healthy controls (blue colour). (**B**) Violin plots showing the expression levels of collagen-related proteins in the corresponding plasma proteomics dataset (left: COL6A1; middle: COL6A2; right: COL6A3). No statistically significant differences were observed for the plasma data. Median values are shown as horizontal lines within the violins.

Analyses were carried out for parent collagen species in the proteomics datasets (both EVs and non-fractionated plasma). The EV proteomic data revealed significantly elevated levels of COL6A3, but not other collagen species, in the participants with impaired vascular health when compared with healthy controls (*P*=0.0091; Figure 10A). In the corresponding plasma datasets COL6A1, COL6A2 and COL6A3 were detectable, but their abundance did not differ significantly between groups (Figure 10B). These data suggest that EVs may provide enhanced sensitivity for detecting ECM-related changes.

As the above differences in EV-derived Col6A3 may arise from differences in the turnover (synthesis versus degradation) of this species, ELISA assays were performed for six collagen-related protein fragments in the full cohort, including the pro-peptide for collagen VI (PRO-C6, which targets the C-terminal end of the COL6A3 chain) as well as the pro-peptide for collagen IV, and degradation markers of multiple collagens. As shown in Supplementary Figure S3A, no statistically significant differences were observed between the healthy and impaired vascular health groups. This finding remained consistent when stratified by sex, with no significant differences detected for either male (Supplementary Figure S3B) or female (Supplementary Figure S3C) subgroups.

**Figure 11:**
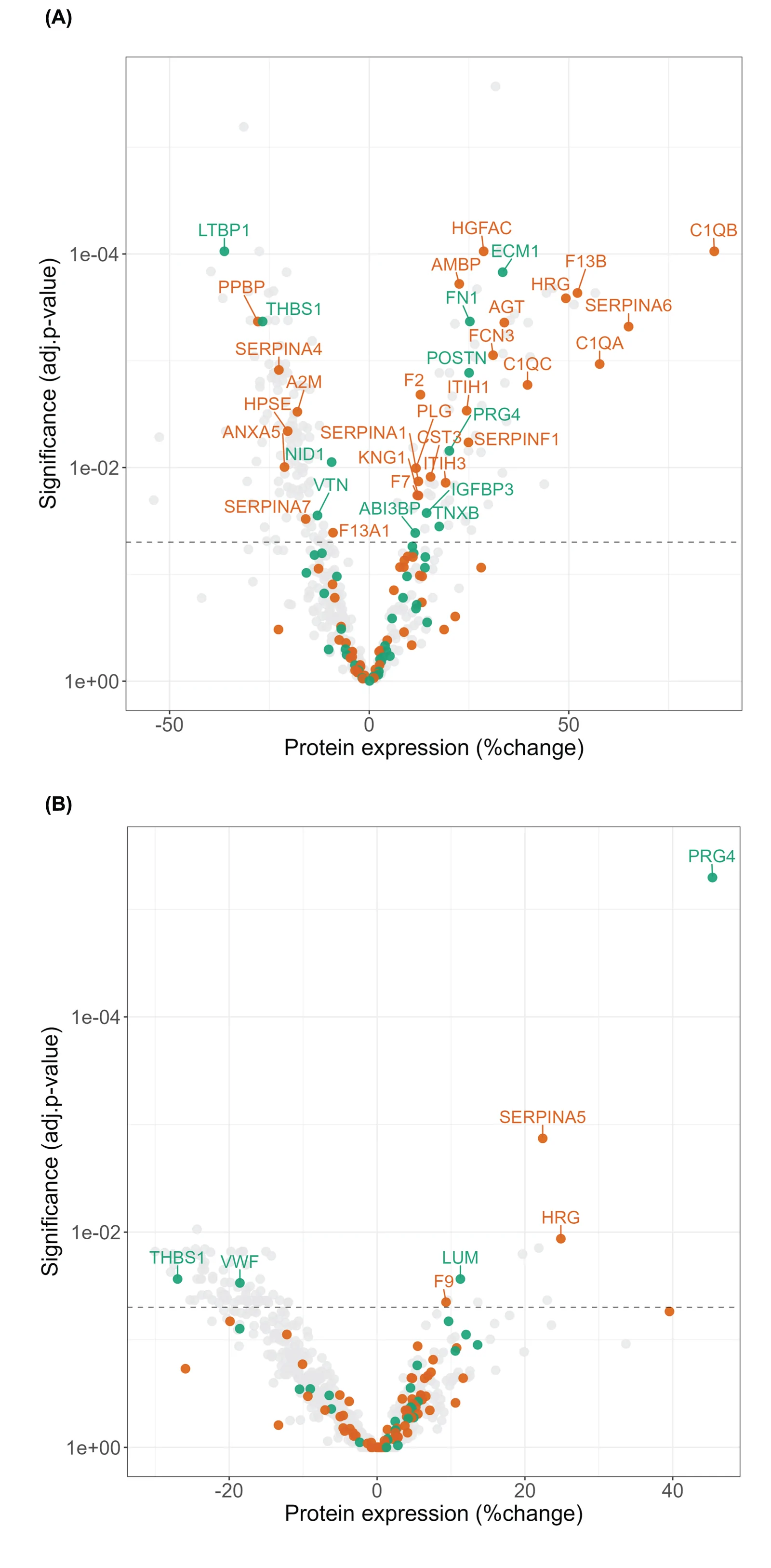
Differential matrisome protein expression in the non-fractionated plasma samples and for participants with impaired vascular health compared to healthy controls Volcano plot of differential matrisome protein expression in the non-fractionated plasma samples (**A**) and EVs (**B**) for participants with impaired vascular health compared to healthy controls. Proteins were annotated based on the MatrisomeDB classification into ‘Core matrisome’ (green colour), ‘Matrisome-associated’ (red colour), and ‘Other’ (grey colour). The horizontal axis indicates the %change in protein expression, and the vertical presents the significance (Benjamini–Hochberg adjusted *P*-value, ‘adj.p-value’ (−log10scale)). Selected matrisome proteins with significant differences are labelled. The dotted horizontal line indicates the significance threshold at adj. *P* = 0.05.

To further investigate ECM remodelling, differential expression analyses were carried out for species defined as ‘matrisome’ species (based on the MatrisomeDB classification [34]) within the EV and plasma proteomes. For the plasma proteome data (Figure 11A), a significant number of core matrisome proteins exhibited significant and high-magnitude changes between groups, indicating a broad and pronounced ECM-related dysregulation. In contrast, the EV proteomic data (Figure 11B) revealed a more selective set of matrisome proteins with moderate fold changes. Notably, COL6A3 was significantly upregulated in EVs from individuals with impaired vascular health but not in plasma, highlighting the potential of EVs to enhance the resolution of disease-relevant ECM markers. Together, these results suggest that plasma provides a more global view of ECM remodelling, while EVs offer refined insight into specific matrix-associated alterations.

## Discussion

In this study, a workflow was developed for the analysis of human plasma-derived EVs. NTA and TEM were used to characterize the size distribution and morphology of particles isolated by the different methods. Although the combination of centrifugation with SEC improved sample cleanliness and reduced co-isolated soluble plasma proteins, this approach resulted in a lower EV concentration with a consequent reduced practicality for working with limited clinical samples, though this approach has been employed and been successful in other studies [42,44]. The lower yield obtained with the combined centrifugation-SEC workflow is likely to be due to cumulative loss of material during repeated centrifugation, pellet resuspension, SEC loading and elution, as well as dilution of EV-associated material across multiple SEC fractions. As a result, the performance of centrifugation and SEC were compared individually (as also reported in [24]). SEC yielded EVs with clearer morphology and a more uniform size as determined by NTA and TEM analyses, whereas centrifugation resulted in a broader particle size distribution (Figure 1). This is likely due to the inclusion of microvesicles and apoptotic bodies [45]. However, for clinical applications, a workflow must balance purity against sample processing capacity (throughput) and efficiency. As comparable proteomic profiles were obtained from both methods (Figure 2), the centrifugation-based protocol was employed for the cohort analyses due to the ease and greater speed of the centrifugation approach, and the ability to process multiple samples simultaneously thereby minimizing handling variables. To further enhance reproducibility, and minimize technical bias, an automated liquid handling system was used for the analyses, with the EV samples randomly assigned to multiple 96-well plates. The same robotic system was also used for cleanup and digestion steps, ensuring consistency throughout the entire workflow. These results support the applicability of this protocol for large-scale clinical sample processing.

Proteomic analysis of the resulting EVs identified 87 proteins with significantly altered abundance between the subjects with impaired vascular health compared to the healthy control group. Enrichment analysis revealed overrepresentation of proteins involved in cytoskeletal regulation and actin binding (Figure 5). These biological processes are essential for vascular remodelling and cellular migration, both of which are central to the development of CVD and atherosclerosis [46]. Several cytoskeleton-related proteins, including filamin, tubulin, and myosin, were differentially expressed and are known to be involved in, and mediate vascular angiogenesis [47–49]. Additionally, a number of ECM and related proteins, such as proteoglycan 4 and lumican, were enriched in the disease group, highlighting the potential role of ECM remodelling in vascular dysfunction and atherosclerotic plaque formation. Coagulation-related proteins were also identified, consistent with an elevated risk of thrombosis in these patients.

Cell-type enrichment analysis confirmed the likely involvement of several key cell populations including platelets, macrophages, neutrophils, monocytes, and smooth muscle cells as probable sources of these species (Figure 5C). Platelets are well-known sources of EVs [20] and facilitate immune cell recruitment during CVD progression. Inflammatory cells such as macrophages, monocytes, and neutrophils contribute to all stages of atherosclerosis by promoting inflammation and plaque formation [50]. Smooth muscle cells, crucial mediators of vascular remodelling, engage in extensive cellular communication during plaque development, endothelial migration, and vascular calcification [51,52].

Biological sex-stratified analyses revealed that the observed protein expression changes were more pronounced in females than males for both the EV and bulk (non-fractionated) plasma (Figures 4 and 6). These findings contrast with previous histological and imaging studies, which often report milder inflammation in female atherosclerosis cases [53]. However, the current data are in alignment with the reported higher incidence of myocardial infarction in women starting from the seventh decade of life [54], though it is acknowledged that the cohorts examined here had limited numbers of subjects in this age category (Supplementary Figure S1). The discrepancy could reflect underlying sex-specific regulatory mechanisms. Alternatively, the attenuated differences in the male group may result from lifestyle factors such as smoking or alcohol consumption, of which only alcohol intake (but not smoking) was higher in the male group (average over 12 months of 11.5 g/day for males, 5.5 g/day for females; estimated from food frequency questionnaires) and could have influenced baseline protein profiles [55]. Thus, of the 51 male subjects, 9 were smokers (18%) and 9 ex-smokers (18%), whereas of the 64 females, 13 were smokers (20%) and 7 ex-smokers (11%). The low numbers in these groups suggest that these data need to be treated with care.

The age-associated analyses of the EVs (Figure 5) identified two proteins—alpha-2-macroglobulin (A2M) and apolipoprotein C-IV (APOC4)—as significantly altered with age in disease versus control groups. In contrast, the bulk plasma proteomic data did not show any significant associations. A2M, a broad-spectrum protease inhibitor, plays a role in coagulation by inhibiting thrombin [56] and has been proposed as a biomarker for CVD [57]. Notably, A2M also exhibits age-independent glycosylation patterns [58], suggesting a potential and unique role in age-related vascular pathology. APOC4, an exchangeable apolipoprotein involved in lipid metabolism, has also been implicated in CVD risk despite its modest plasma abundance [59]. The identification of this species in the current study supports the suggested link between APOC4 and age-related atherosclerotic progression [60].

Comparable proteomic analyses carried out on the non-fractionated plasma from the same subjects also showed significant differences between the two subject groups (Figure 6) confirming the EV data, though the number of statistically significant differences was greater. Gene ontology analyses of these differentially abundant proteins indicated some of the same enriched biological processes and molecular functions, but also additional processes and function as expected. There was, as expected, a significant degree of overlap between the protein identifications in the intact plasma and EV samples (393 common identifications), but only a modest correlation (*R* = 0.55) between these. These was also a significant overlap in the differentially expressed proteins (34 common) and these showed a good correlation with regard to both the magnitude and direction of the observed changes (Figure 9B). However, there were also large numbers of unique proteins in the EVs (104 identifications and 53 differentially expressed species between the two subject groups), indicating that the protein cargo of the EVs is significantly different to that detected in the bulk plasma, and contains significant additional information to that arising from bulk plasma analysis (Figures 8 and 9) and provides potentially important health related data. This is further illustrated by the differences in the biological process enrichment analysis (first two columns of Figure 9A), with many of the terms that were significant for the EVs but not the plasma samples, being related to cellular processes, and particularly cytoskeletal changes and organization. These data support the contention that at least some of the protein cargo present in the EVs reflect changes in cells associated with impaired vascular function.

Previous studies have provided strong evidence for major changes to the ECM of the artery wall during the development of human carotid artery plaques [61], with this including the presence of many protein fragments arising from enzymatic degradation [16]. As a consequence, particular emphasis was placed on possible changes in parent collagens, and species associated with collagen deposition and degradation (e.g., the pro-peptides cleaved removed during synthesis of collagen fibres) in both the EV and bulk plasma proteomic datasets. Whilst no significant changes were detected in the plasma dataset, a significant difference in the level of the collagen VI alpha3 chain (COL6A3) was detected for the EVs, with higher levels detected for the subjects with impaired vascular health (Figure 10A). This enhancement is consistent with the detection of high levels of this collagen isoform (particularly COL6A3, but also the A2 and A1 chains) in atherosclerotic plaques (cf. data in [61]). A number of the other ECM and associated species were also detected as significantly more abundant in the EVs from the impaired vascular health group including PRG4 (proteoglycan 4, also known as lubricin), F5 (Factor V, a key protein in coagulation), the protease inhibitor SERPINA5 [also known as protein C inhibitor (PCI) or plasminogen activator inhibitor 3 (PAI3)] which plays a key role in hemostasis by regulating blood clotting and fibrinolysis, and two apolipoproteins (APOA4 and APOC1) involved in lipid metabolism. Previous proteomic data indicate that these species are present in human carotid artery atherosclerotic plaques [61]. The detection of a number of these species was replicated when a specific search for matrisome proteins was carried on both the non-fractionated plasma and EV datasets (Figure 11), with the levels of proteins associated with a healthy artery wall and decreased platelet aggregation (e.g., von Willebrand’s factor, vWF) detected as overrepresented in the healthy cohort, compared to the impaired vascular health group.

**Figure 12:**
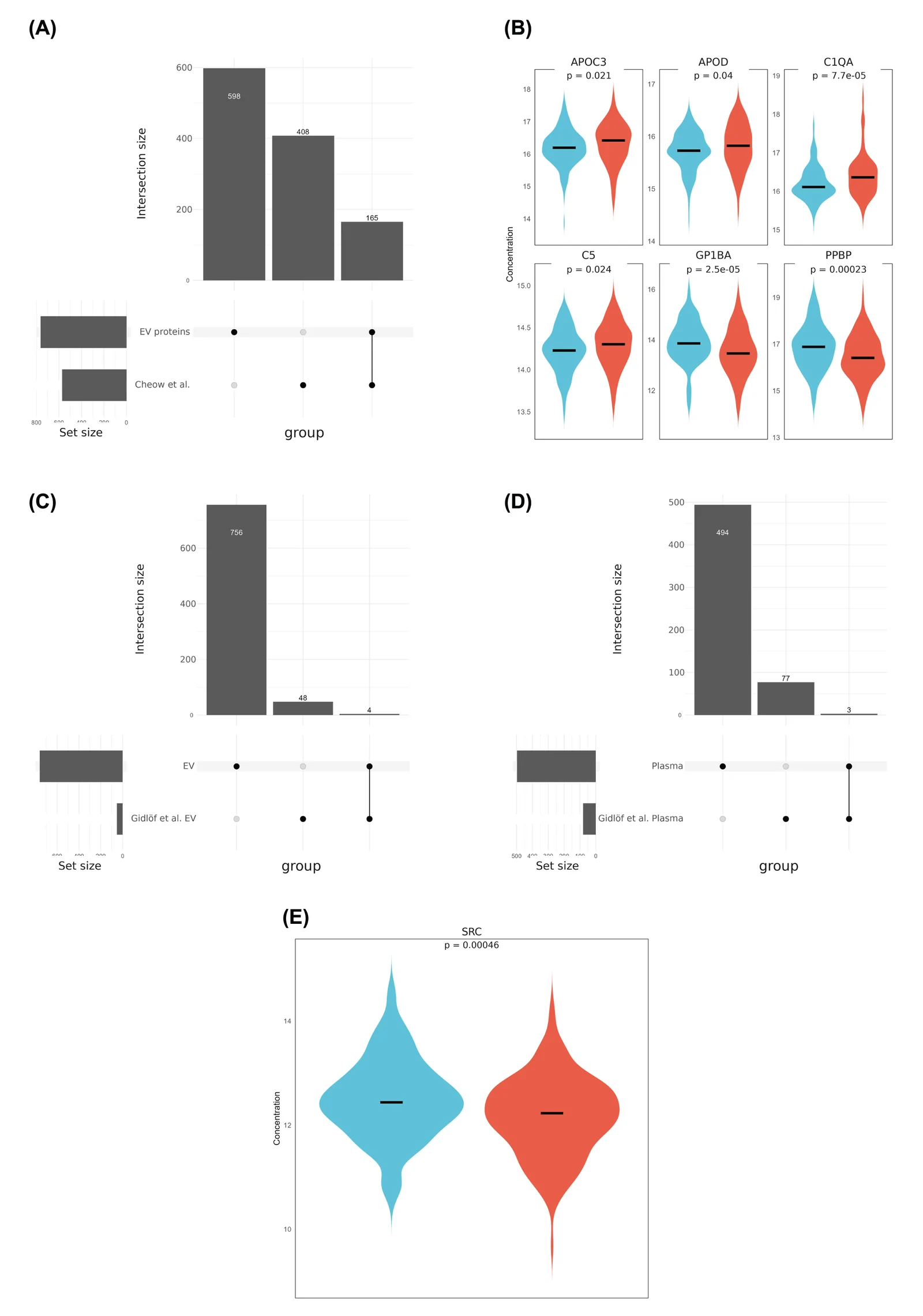
Comparison of EV and plasma proteomes with published datasets (**A**) UpSet comparison of the EV proteome detected in the current study with that reported by Cheow et al. [40] (EV). (**B**) EV abundance data for the current study (red, impaired vascular health; blue, healthy) of the panel of myocardial ischemic (MI)-responsive proteins reported as dysregulated by Cheow et al. [40]; C1QA, Complement C1q subcomponent subunit A; C5, Complement component C5; APOD, Apolipoprotein D; APOC3, Apolipoprotein C-III; GP1BA, Platelet glycoprotein Ib alpha chain (CD42b); PPBP, Pro-platelet basic protein (CXCL7); Wilcoxon rank-sum tests were used to assess statistical differences between the two groups. The horizontal line within each violin indicates the median value. (**C**) UpSet comparison of the EV proteomes detected in this study with that reported by Gidlöf et al. [41]). (**D**) As (C) except for the plasma proteomes. (**E**) EV abundance (red, impaired vascular health; blue, healthy) of SRC (proto-oncogene tyrosine-protein kinase Src) detected in the current study, previously reported as dysregulated by Gidlöf et al. [41]. CTRC and CCl17, also reported by Gidlöf et al. [41], were not detected in the current study.

An UpSet comparison of the current EV proteome data with EV biomarkers reported for a myocardial ischemic (MI) injury cohort [40] showed a shared set of 165 proteins together with a significant number of study-specific detections (Figure 12A). Subsequent analyses were then carried out in the current data for the six EV markers that Cheow et al. reported [40] as higher in the MI group relative to controls (C1QA, C5, APOD, APOC3, GP1BA, PPBP). All six were quantifiable in the current EV data set; the levels of both the complement proteins (C1QA, C5) and the apolipoproteins (APOD, APOC3) showed a similar statistically significant increase in the impaired vascular health group versus the healthy group, as reported in this previous study [40]. However, the two platelet-linked proteins (GP1BA, PPBP) showed a significant decrease in the current data for the impaired vascular health group versus the healthy group (Figure 12B), compared to the increase reported by Cheow et al. [40]. This discrepancy may reflect a difference between the acute nature of ST-elevation myocardial infarction (STEMI) versus the chronic vascular disease in the current cohort. Notably, the exclusion criteria for the BIOCLAIMS Cohort were cardiovascular events (myocardial infarction, stroke, etc.) within 6 months prior to the study, and at the time of investigation, neither clinical symptoms nor clinical chemistry results were indicative of any acute events.

Gidlöf et al. have also reported profiles for EV and plasma proteins using a targeted Olink PEA cardiovascular panel in subjects with STEMI vs. controls [41]. Benchmarking against this previous dataset yielded very limited overlap (4 proteins for EVs and 3 for plasma) consistent with platform and cohort differences (Figure 12C,D). Importantly, when we tested directional concordance for selected STEMI-down-regulated proteins (SRC) reported by Gidlöf [41], the current EV data reproduced the same decrease in the diseased group (Figure 12E), supporting biological consistency across studies despite assay differences.

This study has a number of strengths and weaknesses. Strengths include the well characterized nature of the subject groups (see also [25,62]), the testing and validation of an automated workflow to reduce handling variables, the in-depth proteomic analyses, and the comparison of data from both plasma and EVs from the same subject samples. Weaknesses include the relatively modest numbers of subjects and an incomplete matching of the subject ages between the two groups, due to the fact that subjects of advanced age were less likely to fulfil the strict inclusion criteria of the healthy control group, which may impinge on the biological sex and age-dependent analyses. These limited differences are worthy of follow-up with larger cohorts to address these factors that are known to affect susceptibility to CVD. Further studies should also be carried out in a validation cohort to confirm the changes identified here and extended to other orthogonal techniques (e.g. further ELISA) for validation of the changes in specific protein levels, particularly selected EV-associated candidate proteins, as the current ELISA measurements were limited to selected plasma ECM turnover markers.

## Conclusion

This study has identified a number of biologically relevant proteins from plasma-derived EVs in subjects with impaired vascular function. The results highlight key pathways involved in disease pathogenesis, including cytoskeletal dynamics, ECM remodelling, and coagulation. The ability to trace EV origins to specific cell types suggests the potential of EV profiling as a minimally invasive tool for disease monitoring and early screening. Additionally, our findings on sex- and age-specific expression patterns contribute to a deeper understanding of population differences in atherosclerosis and may guide future personalized risk assessment.

## Supplementary Material

Supplementary Figures S1-S3

Supplementary Tables S1-S7

## Data Availability

The datasets obtained and used in these analyses are available from the corresponding author on reasonable request and in accordance with GDPR regulations.

## Open Access

Open access for this article was enabled by the participation of the University of Copenhagen in an all-inclusive Read & Publish agreement with Portland Press and the Biochemical Society under an agreement with the Royal Danish Library.

## Abbreviations

BMI: body mass index
CAA: 2-chloroacetamide
CAN: acetonitrile
CIMT: carotid artery intimal medial thickness
CVD: cardiovascular diseases
DDM: *N*-dodecyl-β-D-maltoside
eGFR: estimated glomerular filtrate rate
EVs: extracellular vesicles
FA: formic acid
HOMA-IR: homeostatic model assessment of insulin resistance
LC-MS/MS: liquid chromatography-mass spectrometry
MS: mass spectrometry
NTA: nanoparticle tracking analysis
SEC: size-exclusion chromatography
STEMI: ST-elevation myocardial infarction
TCEP: tris(2-carboxyethyl)phosphine
TEAB: triethylammonium bicarbonate
TEM: transmission electron microscopy
TFA: trifluoroacetic acid
