# Supplementary material for: Proteomic analysis of plasma and extracellular vesicles from subjects with impaired vascular health
Source: Biosci Rep. 2026 Aug 25;46(9):BSR20260067. doi: 10.1042/BSR20260067 (PMC13507040; doi:10.1042/BSR20260067)
Supplement: Supplementary file 1 [file BSR-2026-0067-T_supp.pdf]

Supplementary Figures

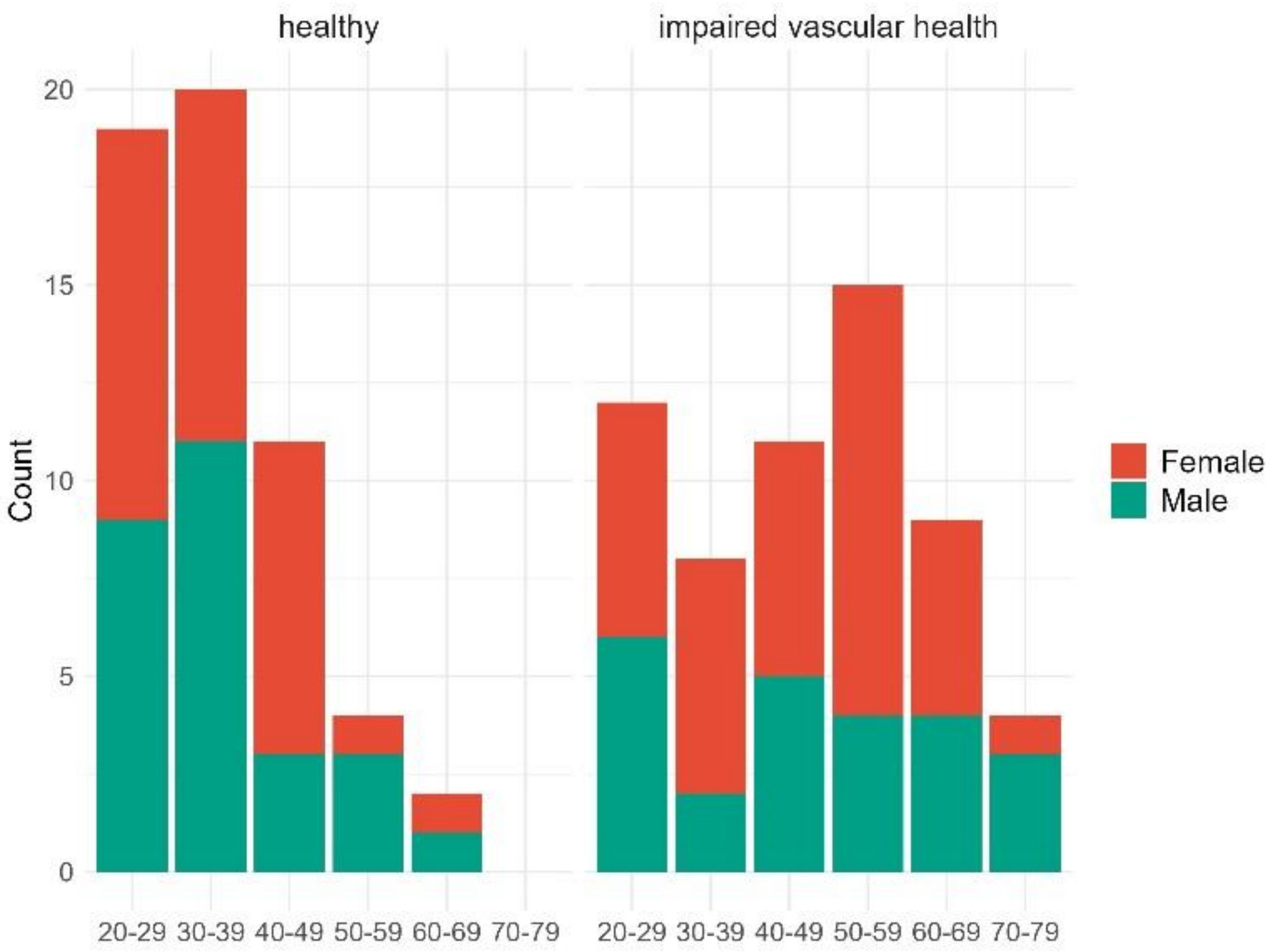

Supplementary Figure 1. Bar plots showing distribution of participants stratified by biological sex (male: turquoise; female: red) and age ranges for the (left side) impaired vascular health and (right side) healthy groups.

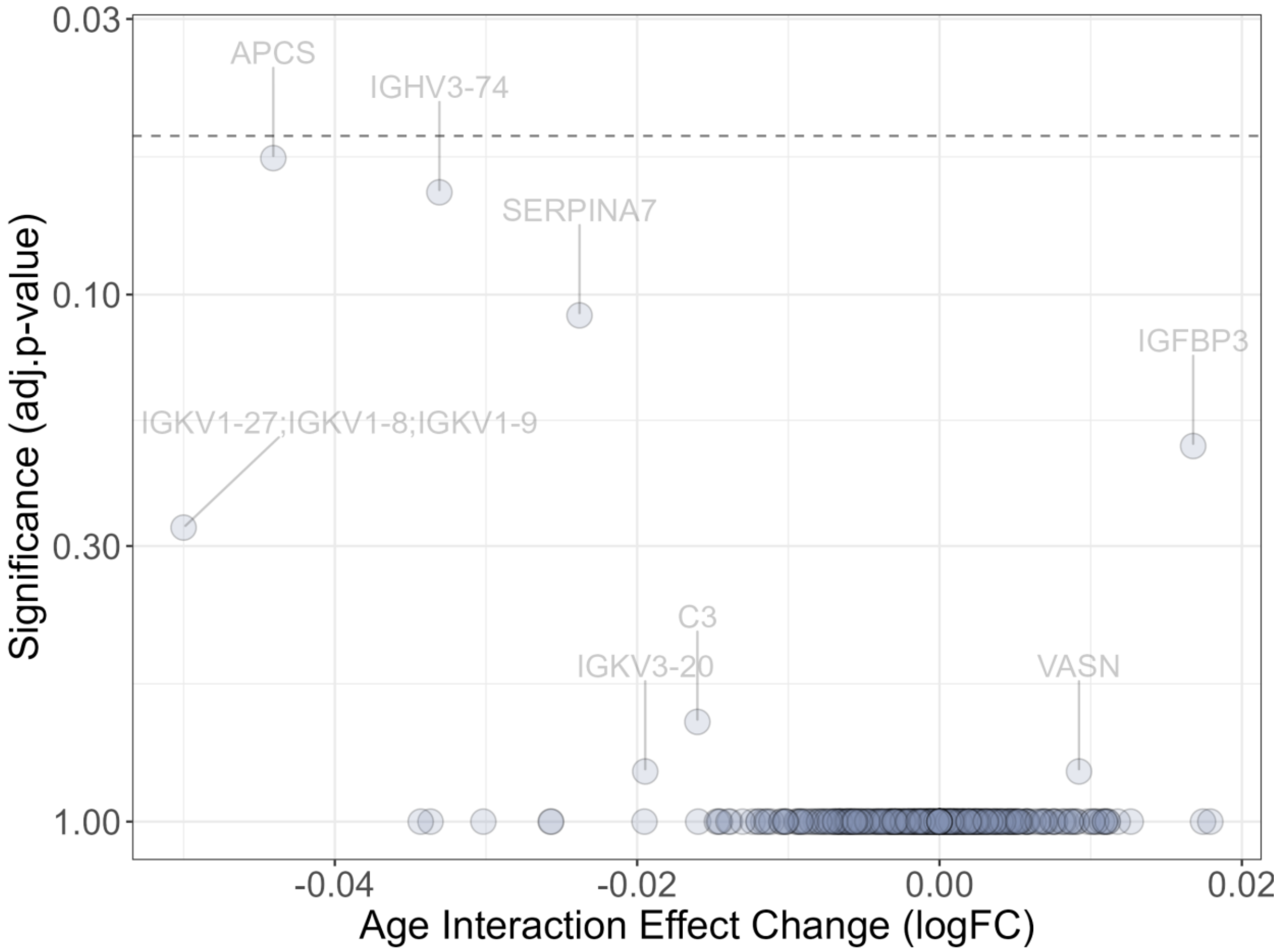

Supplementary Figure 2. Association of differences in plasma protein abundance with subject age for the healthy and impaired vascular health groups.

The x-axis represents the interaction effect size (log fold change) between age and vascular health status, derived from a linear mixed-effects model. The y-axis shows the Benjamini-Hochberg adjusted p-value. The dashed horizontal line indicates the significance threshold at adjusted p = 0.05. Positive values indicate proteins with stronger age-related increases in expression in the impaired vascular health group, while negative values indicate stronger age effects in the healthy group. No statistically-significant age-dependent interactions were detected for the plasma dataset.

(A)

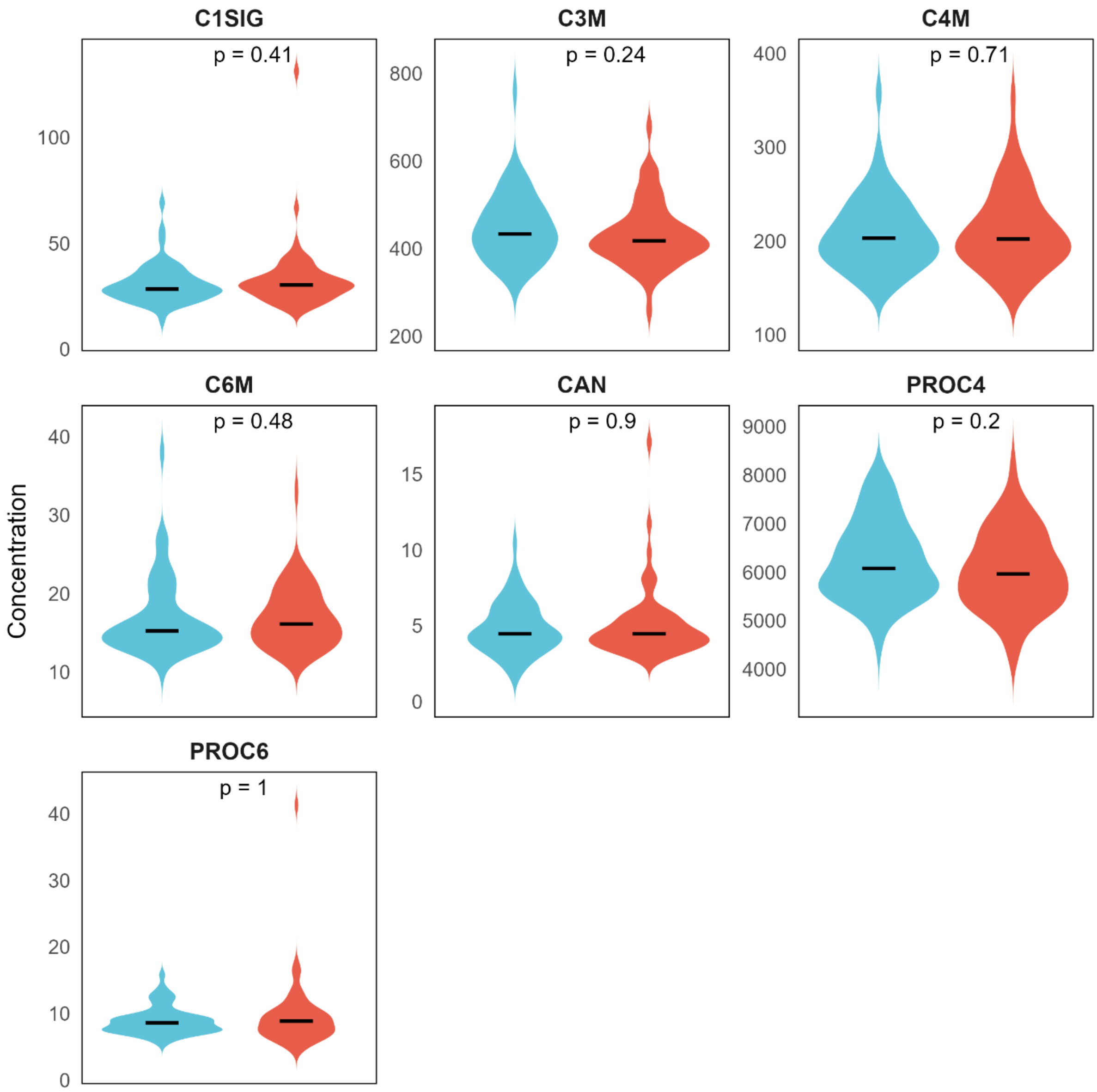

C1SIG
p = 0.41
C3M
p = 0.24
C4M
p = 0.71
C6M
p = 0.48
CAN
p = 0.9
PROC4
p = 0.2
PROC6
p = 1
Concentration

(B)

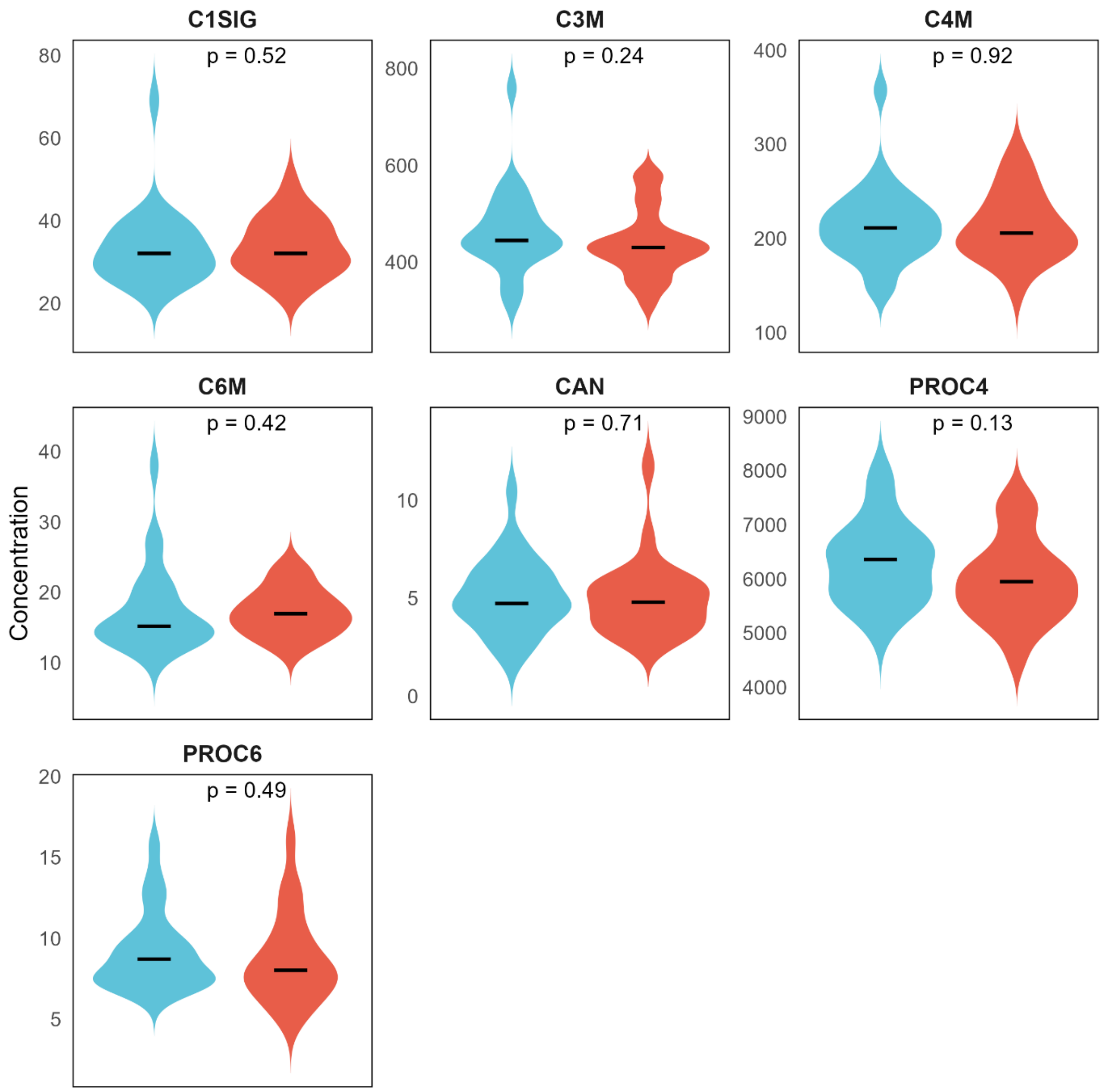
C1SIG
p = 0.52
80
60
40
20
C3M
p = 0.24
800
600
400
C4M
p = 0.92
400
300
200
100
C6M
p = 0.42
40
30
20
10
Concentration
CAN
p = 0.71
10
5
0
PROC4
p = 0.13
9000
8000
7000
6000
5000
4000
PROC6
p = 0.49
20
15
10
5

(C)

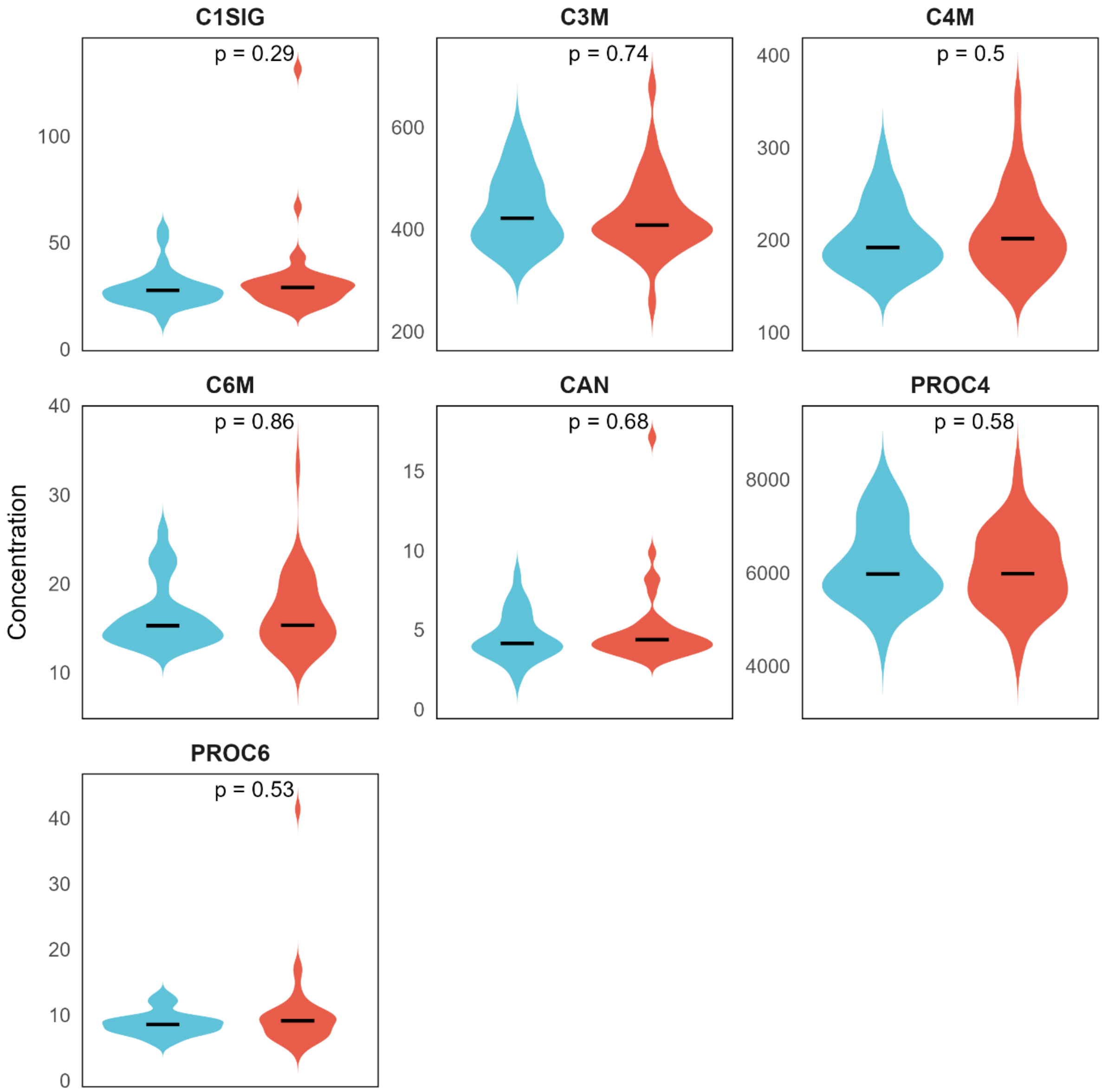

Supplementary Figure 3. Violin plots showing the plasma levels of seven collagen-related fragments, measured by ELISA, in participants with impaired vascular health (red color) and healthy controls (blue color).

Data are presented for the total cohort (A), and the male (B) and (C) female subgroups.

Wilcoxon rank-sum tests were used to assess statistical differences between the two groups.

The horizontal line within each violin indicates the median value. No statistically-significant differences were observed for any of the fragments.

Peptide fragment descriptions: C1SIG, a novel signalling peptide derived from collagen type I; C3M, peptide derived from collagen type III degradation; C4M, peptide derived from collagen type IV degradation; C6M, peptide derived from collagen type VI degradation; CAN, canstatin derived from collagen type IV; PROC4, pro-peptide derived from collagen type IV; PROC6, endotrophin (pro-peptide from collagen VI targeting the C-terminal end of the collagen IV, alpha3 chain).
