# Supplementary material for: Proteomic analysis of plasma and extracellular vesicles from subjects with impaired vascular health
Source: Biosci Rep. 2026 Aug 25;46(9):BSR20260067. doi: 10.1042/BSR20260067 (PMC13507040; doi:10.1042/BSR20260067)
Supplement: Supplementary file 2 [file BSR-2026-0067-T_supp1.zip › BSR-2026-0067-T-s01.pdf]

Supplementary Table 1.

Characteristics of the two study groups (impaired vascular health and healthy subjects) with both statistically-significant and non-significant (NS) data indicated. [1] Non-parametric test (Mann-Whitney) used due to not normally distributed data; [2] *P* > 0.05.

Abbreviations used: BMI, body mass index; IMT, intima-media thickness; FMD, flow-mediated dilatation; BP, blood pressure; LDL, low-density lipoprotein; HDL, high-density lipoprotein; HbA1C, hemoglobin A1C; HOMA-IR, homeostatic model assessment of insulin resistance; GFR-MDRD, estimated glomerular filtration rate using the modification of diet in renal disease formula; ALT, alanine aminotransferase; AST, aspartate aminotransferease; GGT, gamma-glutamyl transferase; LDH, lactate dehydrogenase.

Parameter / units	Impaired vascular health	Healthy	*P* value
Age, y	47.3 ± 15.2	35.3 ± 10.6	< 0.001
Gender, M/F	24/35	27/29	
BMI, kg/m^2	24. 9 ± 3.50	23.1 ± 2.49	0.004[1]
Subcutaneous adipose tissue mass, kg	15.8 ± 5.82	14.2 ± 4.94	NS[2]
Body fat mass, kg	22.1 ± 7.13	18.9 ± 5.47	0.011[1]
Fat-free mass, kg	51.6 ± 10.7	50.8 ± 9.29	NS[1]
Body cell mass, kg	26.8 ± 6.67	27.4 ± 6.11	NS[1]
Phase angle, °	5.90 ± 0.751	6.41 ± 0.676	< 0.001
Waist circumference, cm	88.9 ± 10.6	81.3 ± 8.83	< 0.001
Hip circumference, cm	102 ± 7.29	99.4 ± 6.13	NS
IMT, left, mm	0.649 ± 0.124	0.466 ± 0.071	< 0.001
IMT, right, mm	0.635 ± 0.108	0.482 ± 0.076	< 0.001[1]
Δ FMD, %	10.3 ± 7.50	8.42 ± 5.25	NS[1]
BP, systolic, mm Hg	115 ± 12.1	115 ± 12.2	NS[1]
BP, diastolic, mm Hg	76.9 ± 8.96	74.9 ± 7.31	NS[1]
Cholesterol, mmol/L	5.39 ± 0.831	4.67 ± 0.667	< 0.001
LDL cholesterol, mmol/L	3.27 ± 0.76	2.63 ± 0.61	< 0.001
HDL cholesterol, mmol/L	1.84 ± 0.48	1.75 ± 0.413	NS
Triglycerides, mmol/L	0.81 ± 0.377	0.713 ± 0.268	NS[1]
Apolipoprotein A1, mmol/L	1.69 ± 0.298	1.61 ± 0.237	NS[1]
Apolipoprotein B, mmol/L	0.906 ± 0.202	0.791 ± 0.169	0.0012

Lipoprotein (a), mmol/L	0.608 ± 0.621	0.604 ± 0.742	NS[1]
Glucose, mmol/L	4.90 ± 0.532	4.77 ± 0.419	NS[1]
HbA1C, mmol/mol	37.7 ± 3.06	34.6 ± 3.13	< 0.001
Insulin, mU/L	8.02 ± 3.50	7.55 ± 3.41	NS[1]
HOMA-IR	1.74 ± 0.74	1.61 ± 0.764	NS
Creatinine, mg/dL	0.85 ± 0.132	0.87 ± 0.127	NS
GFR-MDRD, ml/min/1.73 m2	84.8 ± 16.4	87.9 ± 12.4	NS[1]
Cystatin C, mg/L	0.77 ± 0.119	0.755 ± 0.087	NS[1]
Uric acid, mmol/L	312 ± 74.5	302 ± 70.9	NS
C-reactive protein, mg/L	1.42 ± 2.08	1.03 ± 1.11	NS
Fatty liver index	25.3 ± 23.7	12.4 ± 11.9	0.001[1]
ALT, U/L	23.2 ± 13.1	20.4 ± 7.02	NS[1]
AST, U/L	24.8 ± 6.86	22.1 ± 5.10	NS[1]
GGT, U/L	23.7 ± 21.9	17.9 ± 7.34	NS[1]
Alkaline phosphatase, U/L	59.7 ± 21.1	55.6 ± 12.5	NS[1]
Cholinesterase, U/L	7757 ± 1480	7400 ± 1503	NS
Creatine kinase, U/L	127 ± 116	119 ± 67.3	NS[1]
LDH, U/L	168 ± 32.7	168 ± 26.2	NS[1]
Amylase, U/L	28.8 ± 17.0	26.5 ± 7.16	NS[1]
Lipase, U/L	30.8 ± 8.71	30.9 ± 8.72	NS[1]
Albumin, g/dL	4.61 ± 0.209	4.74 ± 0.249	0.0035
Total protein, g/dL	72.4 ± 3.85	74.6 ± 3.85	0.002
